# Predicting the spatial variation in cost-efficiency for agricultural greenhouse gas mitigation programs in the U.S.

**DOI:** 10.1186/s13021-024-00252-6

**Published:** 2024-02-09

**Authors:** Micah V. Cameron-Harp, Nathan P. Hendricks, Nicholas A. Potter

**Affiliations:** 1 Department of Agricultural Economics, Kansas State University, Manhattan, Kansas USA; 2 USDA Economic Research Service, Kansas City, Missouri USA

**Keywords:** Agriculture, Conservation, Policy, Carbon offsets

## Abstract

**Background:**

Two major factors that determine the efficiency of programs designed to mitigate greenhouse gases by encouraging voluntary changes in U.S. agricultural land management are the effect of land use changes on producers’ profitability and the net sequestration those changes create. In this work, we investigate how the interaction of these factors produces spatial heterogeneity in the cost-efficiency of voluntary programs incentivizing tillage reduction and cover-cropping practices. We map county-level predicted rates of adoption for each practice with the greenhouse gas mitigation or carbon sequestration benefits expected from their use. Then, we use these bivariate maps to describe how the cost efficiency of agricultural mitigation efforts is likely to vary spatially in the United States.

**Results:**

Our results suggest the combination of high adoption rates and large reductions in net emissions make reduced tillage programs most cost efficient in the Chesapeake Bay watershed or the Upper Mississippi and Lower Missouri sub-basins of the Mississippi River. For programs aiming to reduce net emissions by incentivizing cover-cropping, we expect cost-efficiency to be greatest in the areas near the main stem of the Mississippi River within its Middle and Lower sections.

**Conclusions:**

Many voluntary agricultural conservation programs offer the same incentives across the United States. Yet spatial variation in profitability and efficacy of conservation practices suggest that these uniform approaches are not cost-effective. Spatial targeting of voluntary agricultural conservation programs has the potential to increase the cost-efficiency of these programs due to regional heterogeneity in the profitability and greenhouse gas mitigation benefits of agricultural land management practices across the continental United States. We illustrate how predicted rates of adoption and greenhouse gas sequestration might be used to target regions where efforts to incentivize cover-cropping and reductions in tillage are most likely to be cost -effective.

**Supplementary Information:**

The online version contains supplementary material available at 10.1186/s13021-024-00252-6.

## Background

In recent years, the United States has experienced an explosion of interest in voluntary programs designed to reduce net greenhouse gas (GHG) emissions by incentivizing changes in agricultural practices. For example, the Inflation Reduction Act specifies that the $8.45 billion appropriated for the USDA Environmental Quality Incentives Program (EQIP) will focus on incentivizing adoption of conservation agriculture practices which increase sequestration or reduce emissions of GHGs [1]. In the private sector, several companies now contract with agricultural producers to generate carbon credits based on the net change in GHG emissions resulting from their land use decisions [2]. Whether public or private, these programs are Payment for Ecosystem Services (PES) programs at their core, and past research on PES programs suggests spatial targeting can greatly improve the cost-efficiency of such programs [3, 4]. For agricultural mitigation programs, cost effective targeting would prioritize areas where the ratio of a practice’s mitigation benefit to its adoption cost, the benefit–cost ratio, is greatest [5]. However, the site-specific data on land management and producers’ adoption costs necessary for benefit–cost targeting are rarely available to policy makers or program administrators.

The benefits of conservation agriculture practices being adopted in a given location are determined by three main drivers of the net change in GHG emissions: climate, soil characteristics, and the current stock of carbon [6]. Of the three, climate is the dominant driver as it determines growing season length, temperature, and precipitation patterns [7, 8]. Sequestration tends to be greater in temperate regions with abundant precipitation as higher temperatures can reduce biomass production and increase the rate of carbon decomposition [9, 10]. Silt and clay content are the most influential soil characteristics determining GHG sequestration because soil and clay can prevent carbon from decomposing by fixing it into mineral-associated forms [11–13]. The final factor, the present stock of soil carbon, results from the interaction between climate, soil, and the history of land management in an area [14–16]. As such, determining the present stock of soil carbon requires largely unavailable records of farming practices like the frequency and timing of nutrient applications and tillage [17–19]. Producers have an incentive to keep such records private as they provide detailed insights into their business decisions within a competitive environment.

Producers have similar incentives to keep the cost of adopting a conservation practice private. If it were available, programs could use this information to pay participants according to their willingness to accept instead of the value of the benefits generated by their participation. In our context for example, a producer may be willing to adopt a land management practice that reduces net GHG emissions for a small incentive because of its beneficial impacts on productivity. While empirical estimates of the cost of adopting agricultural practices that mitigate GHG emissions are available, the results are often specific to the region or sub-samples used in the analysis and may not reflect actual costs to a farm operation [20–23]. Understanding the private costs of adoption can also address the issue of additionality, or the degree to which net reductions in GHG emissions would not have occurred in the absence of the program. Excluding producers who would make the desired land use changes without an incentive, the non-additional adopters, can greatly reduce unnecessary expenditures. For example, a study of the impact of cost-share programs on Iowa producers’ adoption of cover-cropping found 54% of the incentivized cover-crop adoption was additional [24]. A study of producers in Ohio found only a quarter of producers who were paid to adopt conservation tillage were additional due to the practice’s relatively high short run profitability [25], and the estimated rate of additionality at the national scale was 47% [26].

The objective of this work was to demonstrate how targeting voluntary agricultural GHG mitigation programs using aggregate data can make an improvement in cost-efficiency despite the information asymmetry which exists between producers and the policy maker. We begin by presenting a conceptual model of technology diffusion and describe how the relationships we identify between the pace of adoption and additionality inform our empirical approach. Then, after describing the datasets in our analyses, we turn our attention to the method we use to forecast adoption of cover-cropping and tillage practices. In brief, we use a machine learning approach to predict the present rate of adoption and use these predicted rates of adoption to proxy for the expected net return to practice adoption. After combining these predictions with county-level estimates of the net GHG emissions changes associated with each practice, we generate bivariate maps comparing the predicted rates of adoption with expected rates of carbon sequestration.

A related paper by Sperow [27] estimates county-specific carbon prices by assuming county-level EQIP payments equal the cost of adopting various tillage practices. We build on this approach by instead relying on variation in the adoption rate as an indicator of the net return to adoption, the benefits of adoption minus the costs. This has the benefit of addressing within-county variability in the profitability of adoption and the accompanying issue of additionality. As such, our approach relying on the rate of adoption contributes to our understanding of voluntary agricultural mitigation programs by highlighting the role of additionality in determining cost-efficiency.

## Conceptual model and methodology

Our conceptual model is presented in two sections. The first section illustrates how the decisions of agents to adopt conservation agricultural practices generate the county-level adoption outcomes we employ. We demonstrate how the county-level rate of adoption relates to producers’ net returns to adoption, and consequently the likelihood of incentivized adoption being additional. Our conceptual model is inspired by the threshold model of technology diffusion described in [28], the model of voluntary opt-in presented in [29], and the model of additionality in [30]. The second section of our conceptual model adds heterogeneity in the carbon sequestration potential of the practices being adopted. Then, we illustrate our conceptual model of how the interaction between the rate of adoption and carbon sequestration potential of a conservation practice affects the cost efficiency of agricultural mitigation efforts. In the following section detailing our descriptive analysis, we use these same inferences to predict the cost-effectiveness of expenditures on agricultural GHG mitigation programs in meeting carbon sequestration goals in a given county. The numerical example used throughout the conceptual model is meant to illustrate the concepts underpinning our descriptive analysis and not generalized results.

### Model of technology diffusion

Profit-maximizing producers choose whether to adopt a conservation practice in each year, $t\in \left[\mathrm{0,2}\right]$, based on their heterogenous net returns to adoption, ${r}_{i,t}^{c}$, where $i$ is the producer and $c$ is the county the producer is in. The net return to adoption represents the sum of all benefits to using the practice, like yield improvements or reductions in risk, minus the total costs of implementation, such as increased spending on inputs and the opportunity cost of using an alternative practice. A producer adopts the practice if the net return to adoption is positive, ${r}_{i,t}^{c}>0$. The net return to adopting the practice in year $t$ is the sum of a time invariant component reflecting the individual producer’s biophysical and operational characteristics, ${r}_{i}^{c}$ and a time varying component, ${L}_{t}$. The time-varying component to producers’ returns, ${L}_{t}$, represents factors that change over time such as prices, the effectiveness of the practice, and the initial cost of adoption. As such, the distribution of net returns for a producer in a given county, $g\left({r}_{i,t}^{c}\right)$ will shift over time but retain a constant shape determined by the distribution of their producer’s time invariant characteristics, given by $f\left({r}_{i}^{c}\right)\sim N\left(\mu ,1\right)$.

**Fig. 1:**
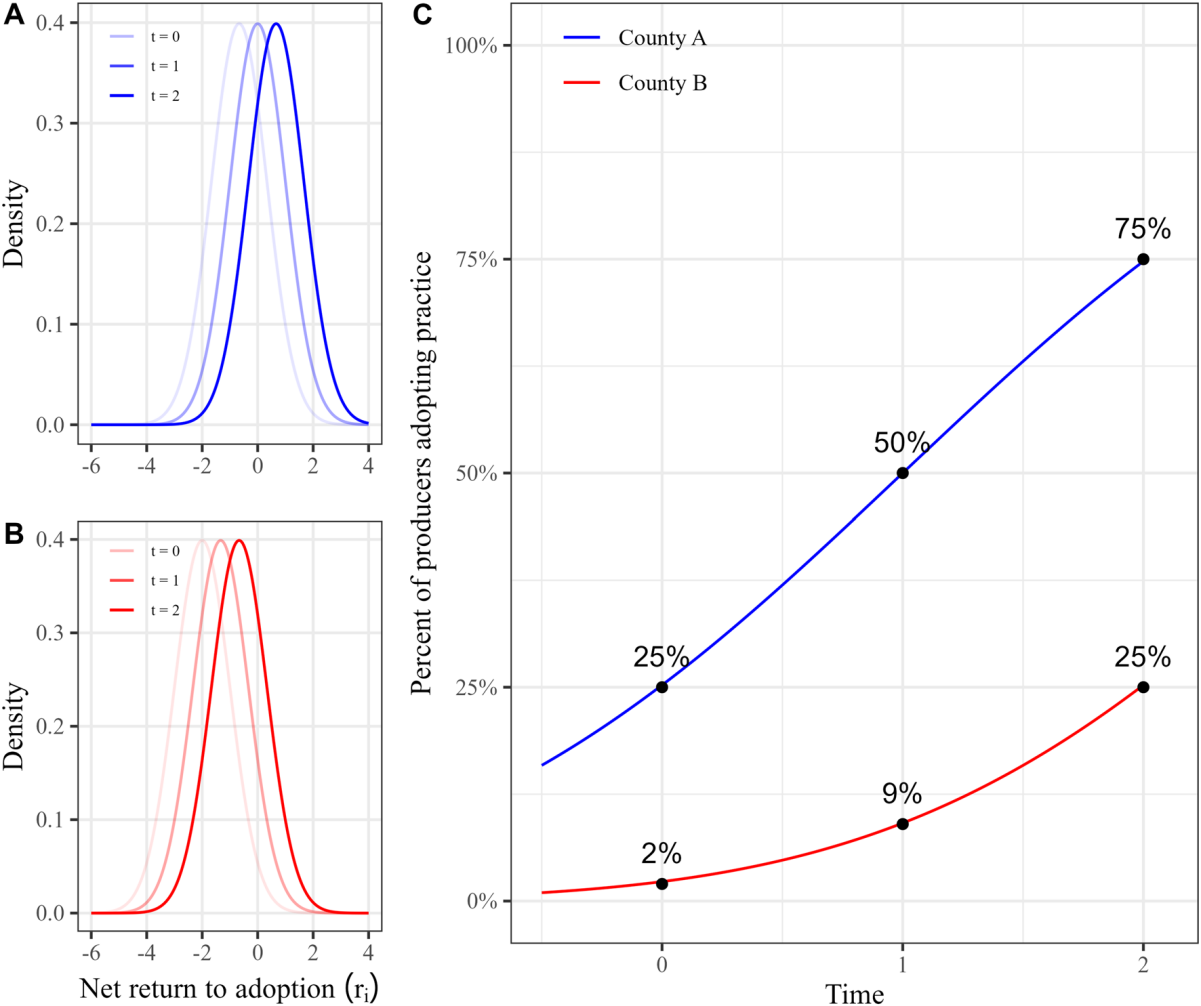
Adoption of conservation practice over time. **A** and **B** depict the probability density functions for the net returns to adoption in counties **A** and **B**, respectively. **C** displays the percent of producers adopting the practice over time for counties **A** and **B**

For example, consider two counties $A$ and $B$ with different time-invariant producer characteristics $f\left({r}_{i}^{A}\right)\sim N\left(-2/3,1\right);f\left({r}_{i}^{B}\right)\sim N\left(-\mathrm{2,1}\right)$. In both counties, the time-varying component evolves identically, ${L}_{t=0}=0;{L}_{t=1}=\frac{2}{3};{L}_{t=2}=\frac{4}{3}$, such that the only difference between the counties is in the mean values of their respective distributions. Panels (A) and (B) of Fig. 1 depict the probability density functions for net returns at times $t=0$ to $2$ in counties A and B, G $\left({r}_{i,t}^{A}\right)$ and G $\left({r}_{i,t}^{B}\right)$. Panel (C) of Fig. 1 depicts the percent of producers adopting the practice over time in counties A and B.

In panels (A) and (B) of Fig. 1, the increase in net returns to adoption caused by the growth of ${L}_{t}$ over time produces the rightward shift of the $G\left({r}_{i,t}\right)$ curves relative to the $G\left({r}_{i,t-1}\right)$ curves. In county A, depicted in the panel (A) of Fig. 1, this shift causes the fraction of the population adopting the practice, $1-G\left({r}_{i,t}^{A}=0\right)$, to double from a quarter to half of the producers between times $t=0$ and $t=1$. For county B, shown in the panel (B) of Fig. 1, an equivalent increase in ${L}_{t}$ between times $t=0$ and $1$ increases adoption from roughly two percent to just over nine percent of producers. Then, after an equivalent increase in ${L}_{t}$ between times $t=1$ and 2, adoption increases by 25 percentage points in county A and 16 percentage points in county B. So, the rate of change in adoption between one period and the next serves as an indicator of the size of the population with net returns near zero, and thus the expected magnitude of adoption in the near future. Furthermore, if we were to continue increasing ${L}_{t}$ and plot the resulting adoption, we would recreate the characteristic “S” shaped adoption curve that typifies the adoption of new agricultural technologies [31–33]. Panel (C) of Fig. 1 depicts these characteristic adoption curves for both counties. Lastly, while we assume a normal distribution for demonstration purposes, these dynamics will hold so long as the distribution of net returns is unimodal [28].

**Fig. 2:**
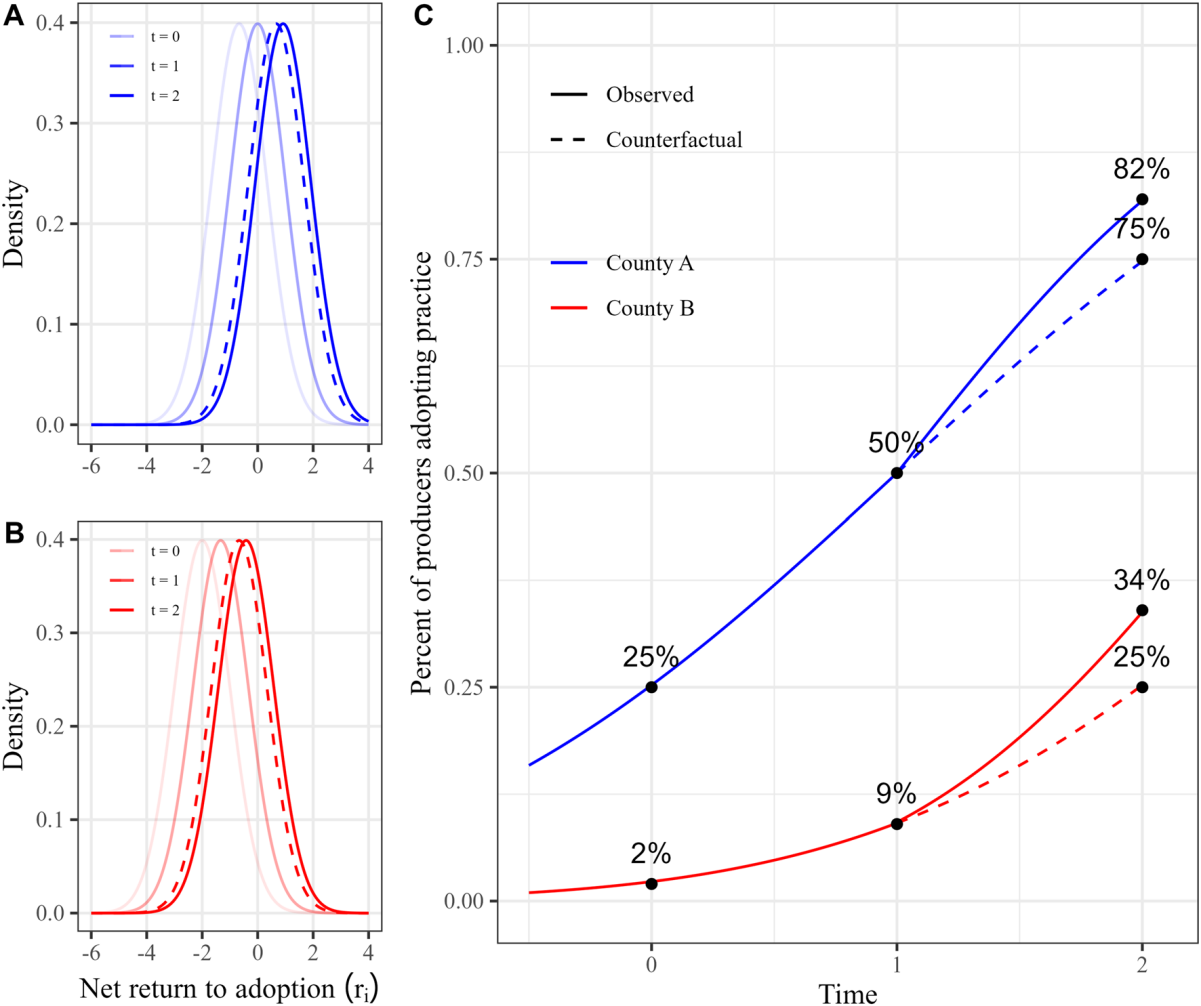
Adoption of conservation practice over time when the payment-for-practice program is available at time $t=2$. **A** and **B** depict the probability density functions for the net returns to adoption in counties A and B, respectively, and **C** displays the percent of producers adopting the practice over time for counties A and B. The dashed graphs indicate counterfactual outcomes without the program

To demonstrate how the relationship between the rate of change in adoption and the size of the population with net returns near zero relates to additionality, consider an administrator operating a payment-for-practice carbon credit program which pays producers a fixed incentive, $${P}_{2}=\$1/4$$, if they adopt the practice between time $$t=1$$ and time $$t=2$$. In addition to the cost of compensating producers, the program administrator also incurs a fixed cost, $${F}_{c}=\$50$$, when establishing the program in a county. The resulting probability density functions are displayed in Panels (A) and (B) of Fig. 2. As depicted in Panel (C) within Fig. 2, an additional seven percent of county A’s producers and nine percent of county B’s producers would enroll and adopt the practice relative to the counterfactual scenario indicated by the dashed curves. The program administrator, however, cannot determine if producers would have adopted the practice in the absence of the incentive without accurately knowing each producer’s individual net return to adoption. As a result, the 25% of producers who would have adopted the practice between times $$t=1$$ and $$2$$ in the absence of an incentive will also enroll in the program, meaning the administrator pays the $$\$0.25$$ incentive to 32% of producers in county A. On average, deploying the program in county A costs the administrator nearly $0.41 per enrollee. But, with only 7% of this adoption being additional, the policy maker pays roughly $1.86 per additional adoptee in county A.

In county B, shown in Panel (B) of Fig. 2, the policy maker pays just under a quarter of producers to adopt the practice, and there are almost twice as many non-additional adopters (16%) as additional adopters (9%). While the payment-per-practice is identical between counties, the average cost per enrollee is greater at $0.45 in county B due to the smaller number of total program enrollees and identical fixed cost. The average cost of an additional adoptee, however, is cheaper in county B at $1.25. This is because in the county with a lower rate of adoption between the first and second periods, a greater portion of the program administrator’s expenditure on incentivizing adoption in the second period goes toward efficient, additional adoption.

In summary, a lower rate of adoption suggests the portion of non-adopting producers in an area with net returns near zero is small and, consequently, programs incentivizing adoption in the region are more likely to create additional adoption. Furthermore, this statement holds when the rate of adoption is negative. For if the county level rate of adoption is negative, the baseline outcome for most producers would be to not use the practice and increase net GHG emissions as a result. As such, incentivizing adoption in areas with negative rates of adoption will reduce net GHG emissions relative to the counterfactual outcome where producers dis-adopt the practice. The crucial assumption needed for comparisons between rates of change to yield valid inferences concerning additionality is that changes in the time-varying component to producers’ returns are the same between counties.

### Model with heterogenous carbon sequestration potential

With the inverse relationship between the rate of adoption and the likelihood of additionality established, we now introduce heterogeneity in the carbon sequestration potential of conservation practice adoption. Consider the same scenario depicted in Fig. 2 except there are now two versions of each county, and the producers in one version of each county sequester more carbon on average if they adopt the conservation practice. These four counties are denoted as ${A}^{low}$, ${A}^{high}$, ${B}^{low}$ and ${B}^{high}$. Counties ${A}^{low}$ and ${A}^{high}$ have the same distributions of net returns as county A in Panel (A) of Fig. 2, and counties ${B}^{low}$ and ${B}^{high}$ have distributions of net returns identical to county B in Panel (B) of Fig. 2. For simplicity, producers in counties with low sequestration potential, ${A}^{low}$ and ${B}^{low}$, sequester 1 ton of carbon if they adopt the practice, and producers in counties with high sequestration potential, ${A}^{high}$ and ${B}^{high}$, sequester 2 tons of carbon. The evolution of ${L}_{t}$, the program administrator’s fixed cost of running the program in each county, and the fixed incentive ${P}_{2}=\$1/4$ for adopting the practice between the second and third periods remain unchanged.

Unlike the earlier program where the goal was increasing adoption of the practice, the objective of the policy maker in this case is to produce carbon credits. The policy maker will generate one carbon credit for each ton of carbon sequestered by a program enrollee. Note that, because the policy maker is still unable to distinguish between additional and non-additional enrollees, it will issue carbon sequestration credits for every enrolled producer. For example, the policy maker would issue 320 credits for the one unit of carbon sequestered by each of the 320 producers in county ${A}^{low}$ who adopt the practice between times $t=1$ and $2$, even though 250 of these producers would have adopted the practice and sequestered 250 tons of carbon even if the program was unavailable. In the same way, the policy maker produces 640, 250, and 500 credits in counties ${A}^{high}$, ${B}^{low}$, and ${B}^{high}$ respectively. The average costs of producing a carbon credit (${AC}_{cred}$) in the four counties, as calculated by the policy maker, are: ${AC}_{cred}\left({A}^{low}\right)=\$0.41;$ ${AC}_{cred}\left({B}^{low}\right)=\$0.45;{AC}_{cred}\left({A}^{high}\right)=\$0.21;{AC}_{cred}\left({B}^{high}\right)=\$0.23$. There are two clear implications for the policy maker. First, in choosing between two counties with the same carbon sequestration potential (${A}^{high}$ vs. ${B}^{high}$; ${A}^{low}$ vs. ${B}^{low}$), targeting the county with a higher rate of practice adoption (${A}^{high}$; ${A}^{low}$) will generate a greater number of credits at a lower per-unit cost. Second, deploying the policy in areas with high sequestration potential is an unambiguous improvement in cost effectiveness:

1 $${{AC}_{cred}(B}^{low})>{{AC}_{cred}(A}^{low})>{{AC}_{cred}(B}^{high})>{{AC}_{cred}(A}^{high}).$$

However, note that the discussion so far has focused only on the cost-effectiveness per carbon credit and not the cost-effectiveness per additional ton of carbon sequestered.

Next, consider the average cost of sequestering an additional ton of carbon, $${AC}_{addseq}$$. Using the $1.86 average cost of an additional adopter for county A from earlier, the average costs of sequestering an additional ton of carbon in the two counties with high rates of adoption are: $${AC}_{addseq}\left({A}^{low}\right)=\$1.86;$$ $${AC}_{addseq}\left({A}^{high}\right)=\$0.93$$. In contrast, the policy maker pays $1.25 and $0.63 per additional unit of carbon sequestered in counties $${B}^{low}$$ and $${B}^{high}$$. Unlike the average cost of producing a credit, the average cost of an additional ton of carbon sequestered is lower for a county with a lower rate of adoption when comparing two counties with identical rates of sequestration:

2 $${{{AC}_{add \;seq}(A}^{low})>{AC}_{add \;seq}(B}^{low})>{{AC}_{add\; seq}(A}^{high})>{{AC}_{cred}(B}^{high}).$$

Additionally, consider a hypothetical county, ${B}^{mid}$, with the same low rate of adoption as counties ${B}^{low}$ and ${B}^{high}$ but having an intermediate sequestration rate of 1.5 tons of carbon. Despite having a lower carbon sequestration rate, the average cost per ton of additional sequestration would be less than that of a county with greater sequestration, ${A}^{high}$:

3 $${{AC}_{add \,seq}(A}^{high})=\$0.93>{{AC}_{cred}(B}^{mid})=\$0.83.$$

So, if using the average cost of additional sequestration as the efficiency metric, targeting areas with the highest rates of carbon sequestration is not always optimal.

**Fig. 3:**
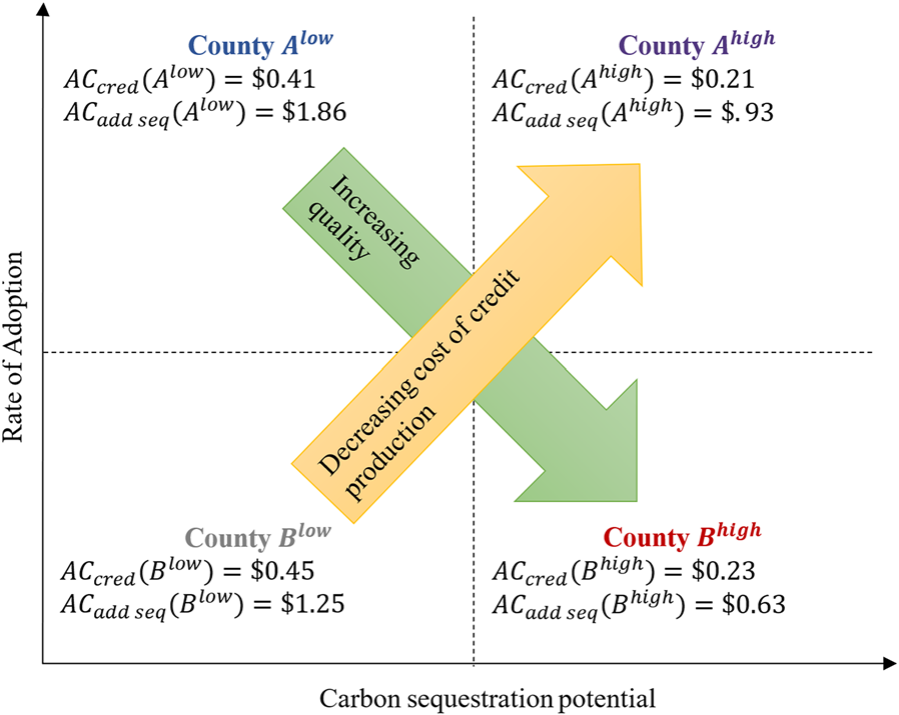
Carbon credit quality and cost as a function of the rate of practice adoption and carbon sequestration in the area where the program occurs

The diagram in Fig. 3 displays the adoption rate and carbon sequestration characteristics for counties ${A}^{low}$, ${A}^{high}$, ${B}^{low}$ and ${B}^{high}$, along with the average cost per credit calculated by the policy maker and the average cost per additional ton of carbon sequestration. To facilitate interpretation of the maps presented in the results section, we label each quadrant of Fig. 3 based on these characteristics. The upper left quadrant of Fig. 3, for example, describes county ${A}^{low}$ with its high rate of adoption and low carbon sequestration potential. In comparison to the lower left quadrant, the lower average cost of a credit indicates producing carbon credits in this county will be relatively cheaper for the policy maker.

If the policy maker sells the credits produced in each county at their respective average costs, then every dollar of carbon credits produced in county ${A}^{low}$ purchased by an entity represents 0.54 tons of carbon sequestered. In comparison to the credits produced in ${B}^{low}$, where every dollar of credits represents 0.8 tons of carbon sequestered, the quality of the credits produced in county ${A}^{low}$ is relatively poor. By making similar comparisons between the remaining counties, we can see that the program administrator’s cost of producing carbon credits is decreasing in the northeasterly direction of Fig. 3, but the quality of the credits produced is increasing in the southeasterly direction. These relationships are the basis for our categorization of counties in the maps we present in the results section.

In Fig. 3, the clear implication of our conceptual model is that targeting areas lying in the eastern quadrants, regions with greater carbon sequestration potential, will generally improve the cost-efficiency of a carbon offset program. Our conceptual model and the associated numerical illustration demonstrate general principles for voluntary carbon sequestration programs, which provide a foundation on which the policy maker can incorporate project-specific information when prioritizing funding. The choice between targeting regions with low or high rates of adoption will of course depend on the specifics of a potential program’s design and the policy maker’s objectives.

## Data

### Adoption of conservation agriculture practices and NRCS programs

The United States Department of Agriculture (USDA) census of agriculture in 2012 and 2017 recorded the number of acres where cover-cropping, no-till, and conservation tillage practices were used in most counties within the continental United States. As we cannot determine whether the change in acreage using no-till is due to producers switching from conventional or conservation tillage, we combine the acreage under no-till and conservation tillage into one category representing the use of any practice with reduced tillage. Lastly, we draw data on total cropland acreage from the USDA National Agricultural Statistics Service (NASS) and use the maximum value recorded for total cropland acres between the 2012 and 2017 data points. To prevent counties with little agricultural acreage from affecting our results, we divide this maximum cropland acres value by the total land area of each county and exclude counties in the lowest decile of the resulting ratio.

To provide a sense of current use of these practices, we generate the share of cropland acres in each practice for 2012 and 2017 by dividing the number of acres in each practice by the maximum total cropland acres value. Additional file 1: Figures S1, S2, and S3 display the change in this share of cropland employing cover-cropping, no-till, and conservation tillage practices over this five-year period. Additional file 1: Figure S4 displays the change in a county’s cropland acreage using either no-till or conservation tillage between 2012 and 2017.

Due to the role policies like EQIP may play in incentivizing adoption of conservation agriculture practices, we include county-level data on the dollars obligated by Natural Resource Conservation Service (NRCS) programs in our analyses [34]. The original data contains all NRCS programs and practices with obligations in the years running from 2014 through 2022. We aggregate these data in three different ways to create annual measures of dollars obligated. The first of these measures is the total across all NRCS programs and practices for each year. We create the second by summing up the obligations for practices concerning cover crops, and the third is the sum across all practices involving a reduction in tillage intensity. We include all three variables because values for some program and county combinations are missing in the original data due to measures taken by NASS to prevent releasing identifiable information. This rarely results in missing values for the measure summing across all programs and practices but creates a greater number of missing values when creating the cover-cropping and tillage specific aggregations.

### Carbon sequestration potential of practices

The COMET Planner tool contains county level estimates of the net sequestration, or total change in GHG emissions, caused by NRCS Conservation Practices [35]. Within COMET Planner, the values for a specific implementation of a NRCS Conservation Practice Standard (CPS) are aggregations of estimates produced using the COMET Farm tool, a field-scale platform for estimating carbon fluxes using the DayCent process-based model [36]. To create a single estimate for the adoption of no-till or conservation tillage practices, we take the mean of all county-level estimates for a change from intensive tillage to reduced tillage (CPS 345) and a change from intensive tillage to no-till or strip-till (CPS 329). The mean expected sequestration in metric tons of carbon dioxide equivalents per acre per year for the practices is displayed in Additional file 1: Figure S5.

To generate the expected carbon sequestration associated with cover-cropping, we take the mean of COMET Planner estimates for scenarios under USDA-NRCS CPS number 340. These scenarios include the addition of a legume seasonal cover crop with a 50% reduction in nitrogen fertilizer or the addition of a non-legume seasonal cover crop with a 25% reduction in nitrogen fertilizer. The mean expected sequestration for cover-cropping is depicted in Additional file 1: Figure S6. In creating the average sequestration values for a reduction in tillage or adoption of cover crops, we do not include scenarios where multiple practices are adopted jointly. For instance, we do not include scenarios involving the joint adoption of no-till and cover-cropping contemporaneously when generating the expected carbon sequestration associated with tillage reduction in a county. When combining the carbon sequestration data with the rates of adoption to create the final maps displayed in the results section, the carbon sequestration values are divided into three categories by tercile.

### Soil, climate, and weather characteristics

Each county’s time-invariant soil characteristics, such as water holding capacity, are drawn from the Soil Survey Geographic Database (SSURGO) [37]. To ensure the values for each soil characteristic reflect cropland soils, we first filter out areas which were not identified as cultivated cropland using the Cultivated Layer data from the 2013 and 2017 Cropland Data Layers [38]. We then overlay these maps of cultivated cropland with a grid of one square mile cells, remove any cells that are less than 50% cultivated cropland in both layers, and use the soil map unit keys from the remaining cells to retrieve the respective soil characteristics. As the Cultivated Layer identifies areas which were cultivated in at least two of the five years preceding the specified year, this process ensures the SSURGO data are drawn from cropland areas cultivated for at least two of the five years before each of the USDA censuses in 2012 and 2017.

For the climate and weather variables, we focus on the two main determinants of carbon sequestration described above: temperature and precipitation. Daily data on counties’ rainfall, maximum temperature, and minimum temperature were drawn from the Parameter-elevation Regressions on Independent Slopes Model repository maintained by Oregon State University [39]. For precipitation, we aggregate the daily rainfall data into pre-season, growing-season, and post-season totals based on whether the rainfall event occurred between the beginning of January and end of February, between the beginning of March and the end of August, or after September 1st respectively.

To aggregate the temperature data, we first create exposure variables as in Schlenker and Roberts (2009). Specifically, the first exposure variable counts all days with temperatures below 0 degrees Celsius. The second, third, and fourth bins represent the days spent in each of the three 10-degree intervals between zero and 30 degrees Celsius. Finally, the last temperature exposure variable represents extreme heat days and contains the days of exposure to temperatures above 30 degrees Celsius. As with the precipitation data, we create total temperature exposure variables representing the days spent in each respective bin during the pre-season, growing-season, and post-season months described above.

The climate characteristics used in our predictions are the average of the temperature and precipitation variables across the 20 years before each NASS datapoint. The weather characteristics, in contrast, are the average deviation from these 20-year averages, or normals, during the five-year periods between 2012 and 2017 or 2017 and 2022. We calculate the deviation in each year by subtracting the annual data from the respective 20 year normal.

## Methods

### Predicting the rate of practice adoption between 2017 and 2022

As described in the previous section, the Census of Agriculture provides data on the historical rate of practice adoption. However, our objective is to parameterize the future rate of practice adoption because it predicts the additionality of programs encouraging conservation agriculture practices. We use a random forest model trained on historical data from 2012 and 2017 to predict the change in reduced tillage and cover cropping adoption from 2017 to 2022. Random forests are a type of machine learning algorithm that averages the output from multiple decision trees. A random forest model is superior to a logistic or linear regression for prediction because they provide improved predictive accuracy, accommodate highly non-linear relationships between predictors, do not rely on parametric assumptions, and incorporate assessment of out-of-sample prediction error [41, 42]. We use the generalized random forest algorithm by Athey et al. (2019) to generate predictions for the rate of change in each practice’s use. Like the original random forest by Breiman (2001), the generalized random forest algorithm involves subsampling the dataset, recursively partitioning the sample into training and test sets, and randomly selecting variables to split the sample. To mitigate bias in predictions, the generalized random forest algorithm from Athey et al. (2019) trains ‘honest’ forests such that separate subsamples are used to determine the optimal splits for each tree and make predictions [45].

To construct the training dataset, we merge the soil, weather, and growing region variables with the observed adoption behavior in 2012 and 2017 from the USDA Census of Agriculture. The objective of the training exercise is to minimize the squared prediction error when modeling the following relationship:

4 $$ln\left(\frac{{y}_{i,t}}{{y}_{i,t-\tau }}\right)=f\left(\frac{{y}_{i,t-\tau }}{{cropland}_{i}},ln\left({y}_{i,t-\tau }\right),{X}_{i,t}\right);\tau =5,$$

where ${y}_{i,t}$ is acres using a conservation practice in county $i$ for year $t$, ${cropland}_{i}$ is the log transformed maximum of the 2012 and 2017 cropland acres for county $i$, and ${X}_{i,t}$ contains the growing condition variables. Within ${X}_{i,t}$ are the temperature and precipitation normals, the average deviation from the normals over the five years preceding $t$, USDA-ERS farm resource region indicator variables, the measures of dollar obligations for NRCS programs, and time-invariant soil characteristics.

The interval between time points, $\tau =5$, in Eq. 4 reflects the gap between USDA Census datapoints. We take the mean deviation from the precipitation and temperature normals across these five years because we do not know the year when adoption of a practice occurred. So, as we only know if adoption occurred within the five-year period, we allow weather conditions in any of the intervening years to affect adoption equally. Similarly, we use the mean values for the annual NRCS program obligation measures from 2014 through 2017 when fitting the random forest using the observed practice adoption data and use NRCS program obligation measures from 2017 to 2022 when predicting the rate of adoption between 2017 and 2022. In addition, to account for differences in county size, we divide the NRCS obligation measures for each county by the county’s cropland acres.

To generate the random forest, we use the *grf* package by Tibshirani et al. (2023) which implements the algorithm defined by Athey et al. (2019). We grow the random forest to have 2000 trees and allow the *grf* package to select the optimal parameter values for the *sample.fraction*, *mtry*, *min.node.size*, *alpha*, and *imbalance.penalty* parameters. We set the *honesty.fraction* parameter so 80% of each sample is used to determine the optimal splits in trees and the other 20% is then used to generate predictions. Due to the large number of predictors, we train an initial forest on all the variables, and then train our final forest using only the variables most frequently used to make splits [47]. This iterative forest procedure can improve the predictive performance of random forests when there is a low signal to noise ratio in the data [48]. The optimized parameter values for the random forests produced using this iterative procedure are listed in Table 1.

**Table 1:** Parameter values for the random forests predicting the change in acres using tillage reduction and cover-cropping practices

	Parameter values				
	*Sample.fraction*	*mtry*	*min.node.size*	*Alpha*	*Imbalance.penalty*
Tillage reduction	0.38	10	3	0.08	0.72
Cover cropping	0.45	7	7	0.14	0.36

To evaluate the quality of our random forests, we report two measures of fit in Table 2 that are generated by the *grf* package during the training exercise using the 2012 and 2017 data. The first measure indicates if the mean forest prediction is correct, while the second measures the degree to which the random forest accurately reproduces heterogeneity. For both tests, a value of 1 indicates the well forest is well calibrated. To determine whether the random forest performs poorly in particular regions, we display the variance estimates for our predictions in Additional file 1: Figures S7 and S8. The asymptotic theory informing the variance estimates is presented by [49] in their analysis of regression forests as *U*-statistics.

**Table 2:** Tests of the random forests predictive accuracy using the held-out, or out-of-bag, data from the sub-sampling procedure in each iteration

	Estimate	Std. Error	t-value	Pr(> t)
Tillage reduction				
Mean forest prediction	1.01	0.06	15.91	< 0.001
Differential forest prediction	1.17	0.09	13.15	< 0.001
Cover-cropping				
Mean forest prediction	1.01	0.04	25.37	< 0.001
Differential forest prediction	1.13	0.04	26.44	< 0.001

After training the random forest using the county-level data on use of tillage reduction and cover-cropping practices from 2012 and 2017, the rate of change between 2017 and 2022 is then predicted using the same set of predictor variables. The resulting county-level rates of change in practice use, expressed as the natural log of the ratio between acres using the practice in 2022 and 2017, are displayed in Additional file 1: Figures S9 and S10 for tillage reduction and cover-cropping practices respectively. To clarify the relationships driving these predictions, Additional file 1: Figures S11 and S12 display the relative importance for the variables selected in the iterative forest procedure. The most important predictor of the rate of change in both tillage reduction and cover-cropping practice use is the lagged adoption rate.

The results, displayed and discussed in the next section, are presented as two-way choropleths. To produce the choropleths, we divide the predicted rates of change from the random forest into three intervals and match them by county to the terciles of carbon sequestration rates contained in COMET Planner. For the predicted rates of change in practice use, the first interval contains all counties with negative predicted rates of change, and the second and third intervals contain counties with rates of change below and above the median positive value. Additional file 1: Figures S13 and S14 display the intervals for the predicted rates of change in practice use and terciles for sequestration rates associated with tillage reduction practices as an example. In our conceptual model, we used two categories of sequestration and adoption rates to illustrate general principles in a simplified context. For our results, we use three intervals to provide a richer depiction of the heterogeneity between counties. In practice, policy makers and program administrators could divide sequestration and adoption rates into a greater number of intervals or use the raw values to prioritize regions more precisely.

## Results

We focus our analysis on four categories of counties defined by their predicted rates of adoption and carbon sequestration potential. Using these categories, we analyze the cost efficiency of expenditures on voluntary agricultural GHG mitigation programs at the county level across the continental United States. Counties in the first category have negative predicted rates of adoption and net sequestration values in the lowest tercile. The negative rates of adoption suggest producers in this first category of counties will require large incentives to adopt, and the low net sequestration values indicate the benefit of their adoption will be minimal. The second category is defined by high predicted rates of adoption but low carbon sequestration. While producers in these counties may require a smaller incentive to adopt conservation tillage, they are also more likely to be non-additional. Counties in the third category comprise the opposite case with negative predicted rates of adoption and high carbon sequestration potential. Producers in this third group of counties may be less likely to adopt, but adoption will lead to larger per adopter reductions in carbon emissions on average. When deciding how to allocate funds between counties in the second and third categories, policy makers will need to consider these trade-offs given the lack of information on precisely how many adopters will be non-additional to accurately estimate the benefit–cost ratio. Alternatively, policy makers could evaluate the cost of using a more complex program design to address additionality. The final category of counties is characterized by high predicted rates of adoption and carbon sequestration, which offer a cost-effective combination of both high adoption and high sequestration.

### No-till, conservation tillage, or reduced tillage practices

**Fig. 4:**
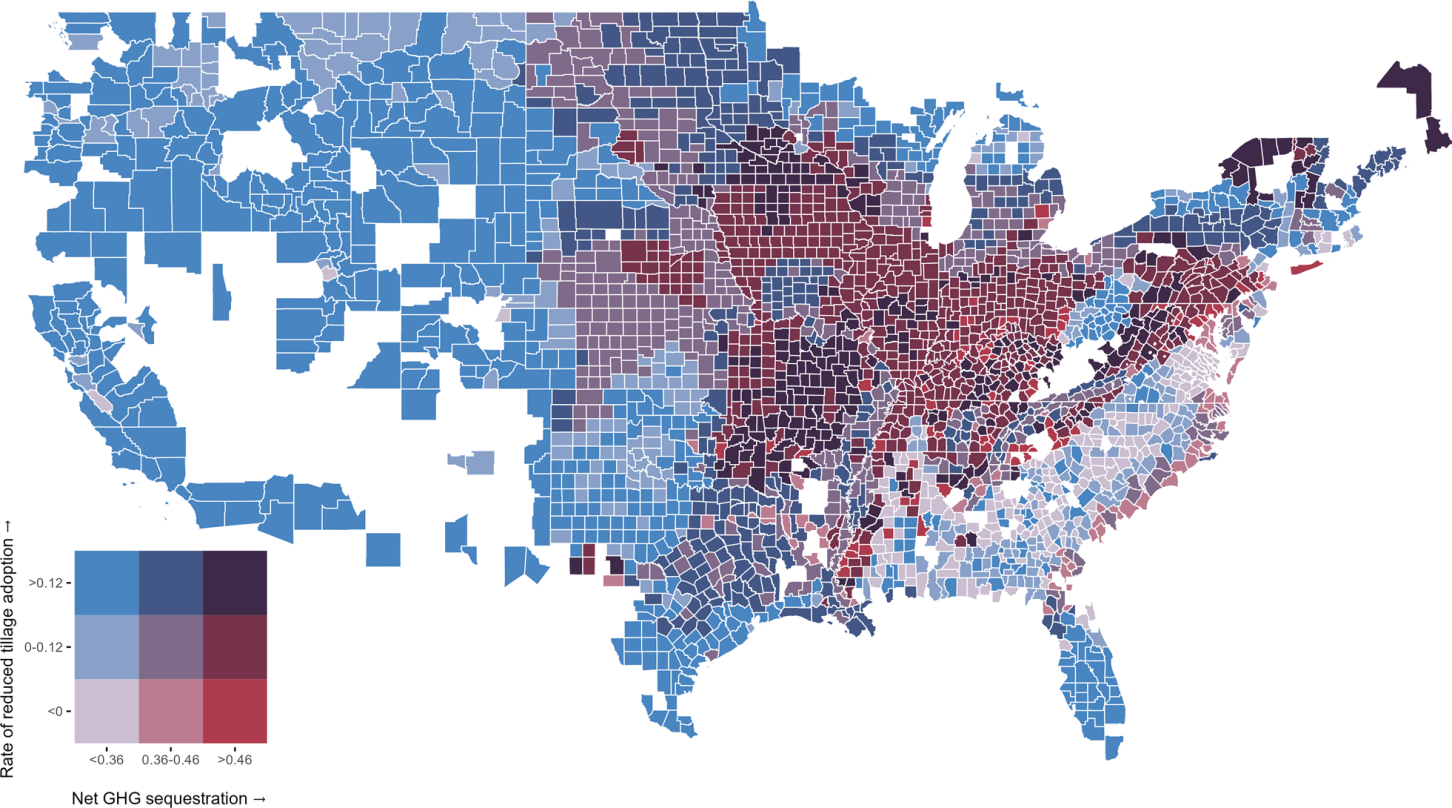
Two-way choropleth depicting the predicted rates of adoption for tillage reduction practices with the net sequestration due to their use. Net sequestration values are divided into terciles and expressed in metric tons of carbon dioxide equivalents per acre per year. Rates of adoption are expressed as the natural log of the ratio of acres using the practice predicted for 2022 to the acreage recorded for 2017. Counties with missing data are in white

In Fig. 4, we display the predicted rate of change in use of reduced tillage practices between 2017 and 2022 along with the rate of greenhouse gas sequestration at the county-level as a bivariate choropleth. Darker blue counties have greater rates of predicted adoption, indicated by moving upward in the legend. Darker red counties sequester more carbon from adopting the practice, indicated by moving rightward in the legend. Counties in white are missing data on conservation agriculture practice adoption for 2017.

First consider the counties in light purple, those in the first category with negative rates of adoption and net sequestration values in the lowest tercile. These counties are predominantly located east of the Appalachian Mountains within the Atlantic Plains stretching from Delaware to Florida. Using the relationships from our conceptual model, we classify agricultural GHG mitigation programs in these areas as inefficient. The negative rate of adoption suggests it will take a large incentive for producers to change their practices, and the marginal increase in carbon sequestration for each producer who does make the change is quite small.

Many of the semi-arid and arid states in the west contain counties with low sequestration rates but high rates of adoption for tillage reduction practices, indicated by the bright blue color in the top left of Fig. 4’s legend. In addition, there are also pockets of such counties in the Great Lakes region, Texas, and Florida. While encouraging adoption in these areas is relatively cheap due to the high predicted rate of adoption, the cost-efficiency of agricultural mitigation efforts will be inhibited due to the combination of low expected sequestration rates and high likelihood of non-additional adoption.

Counties in bright red in Fig. 4, with negative predicted rates of adoption but expected sequestration in the highest tercile, are concentrated in two areas: sub-basins of the Mississippi River system and the Chesapeake Bay watershed. Given the negative rates of adoption, producers will likely require a large incentive to adopt or continue using practices with reduced tillage intensity. However, the producers who are incentivized to reduce their tillage intensity are likely to be additional. So, while encouraging adoption may be somewhat expensive, the expenditures are likely to induce additional adoption behavior or avoid emissions by prolonging the use of practices by producers who would have dis-adopted them otherwise.

The final category of counties we focus on, those in dark purple in Fig. 4, have a high predicted rate of adoption and high expected carbon sequestration for tillage reduction practices. These counties are scattered throughout the Mississippi river basin, the Chesapeake Bay watershed, and the northern portions of Maine and New York. Due to their high adoption rate, incentivizing producers to adopt tillage reduction practices would be inexpensive. While this high rate of adoption suggests a reduced likelihood of additionality, the high expected carbon sequestration associated with adoption in these regions serves to counterbalance the inefficiency introduced by non-additional adopters. Taken together, operating a program designed to reduce net GHG emissions through agricultural land management in these areas is likely to be a cost-efficient endeavor.

### Cover-cropping

**Fig. 5:**
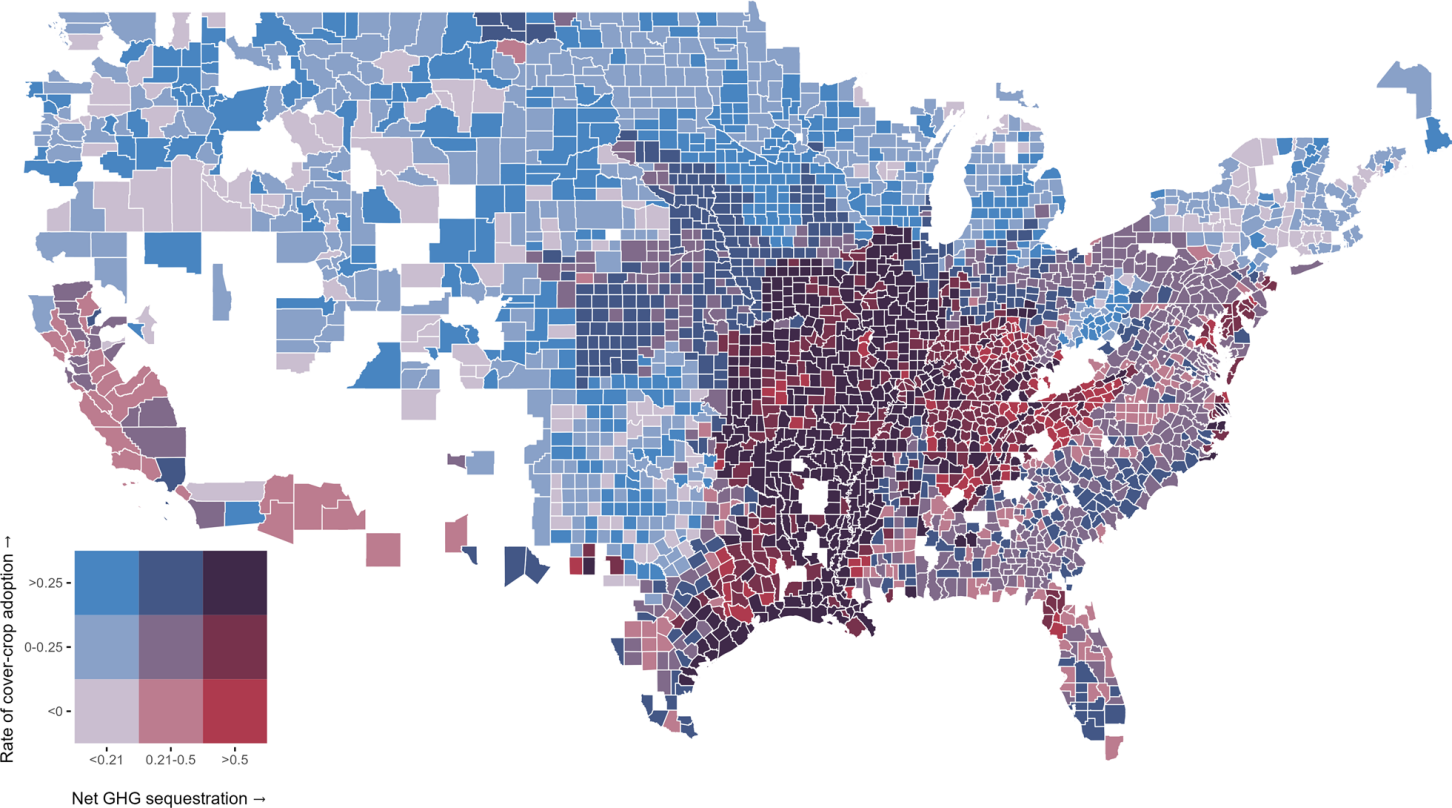
Two-way choropleth depicting predicted rates of adoption for cover-cropping with the expected sequestration due its use. Net sequestration values emissions are divided into terciles and expressed in metric tons of carbon dioxide equivalents per acre per year. Rates of adoption are expressed as the natural log of the ratio of acres using the practice predicted for 2022 to the acreage recorded for 2017. Counties with missing data are in white

Figure 5 depicts the predicted rate for cover-crop adoption between 2017 and 2022 with the associated rate of greenhouse gas sequestration using the same relationships and categorizations outlined above for Fig. 4. Given the additional water demand cover-crops represent, the greater occurrence of counties with negative predicted rates of adoption and sequestration in the arid and semi-arid regions of the western United States is not surprising. If rainfall and access to irrigation are insufficient to support cover-cropping, producers will require larger incentives to incorporate cover-cropping into their land management. In general, as is the case for tillage reduction practices, voluntary agricultural mitigation programs focused on cover-cropping would be expensive efforts producing little in the way of sequestered carbon or avoided emissions in many western states.

The greatest concentration of counties with high predicted rates of change and low expected sequestration for cover-cropping is in the northern Midwest and spans parts of Minnesota, North Dakota, and South Dakota. There are also counties scattered throughout the western United States and around the Great Lakes that share these characteristics as well. Due to the low expected sequestration and high predicted rate of change in cover-cropping, programs in these regions are less likely to be cost-effective. Adoption is more likely to be non-additional, and the marginal change in net GHG emissions for any adoption which is additional will be small in magnitude.

Next, we focus on the counties in our third category in Fig. 5, those with high expected rates of carbon sequestration, or avoided emissions, and negative rates of change for cover-cropping practices. Most of the counties with high expected rates of sequestration and negative predicted rates of change in cover-cropping use, in bright red in Fig. 5, are located near confluences of the Mississippi River and its five major tributaries. The exceptions to this statement are the cluster of counties in the southern portion of the Ohio River basin and the group of counties in south-eastern, coastal Texas. If programs are especially concerned with minimizing non-additionality, these regions will be the natural areas to target provided the program administrators are willing to pay the steep incentives necessary to encourage cover-cropping.

Similar to the previous category, counties in the final category with high predicted rates of adoption and high rates of carbon sequestration for cover-cropping are concentrated along the main stem of the Mississippi River or its major tributaries. The increase in expected sequestration or avoided emissions in riparian areas is due to cover-crops reducing soil erosion and nutrient pollution [35]. Again, as was the case for tillage reduction practices, these regions are likely to be the most cost-effective regions to target. While adoption may be non-additional, the risk of non-additionality is offset by the small incentives required to change behavior and the large reductions in net GHG emissions.

## Discussion

The results of this study demonstrate how publicly available data on county level adoption of conservation practices can be combined with expected sequestration rates to target expenditures on agricultural GHG mitigation programs. Policy makers and private companies implementing such programs can use these results and the general approach to prioritize regions without having to acquire data on individual producers. One immediately apparent similarity between the results for tillage reduction practices in Fig. 4 and the results for cover-cropping depicted in Fig. 5 is the prevalence of counties with negative predicted rates of adoption and low expected carbon sequestration in the arid and semi-arid regions of the western United States. Given the high cost of encouraging adoption and little return in terms of carbon sequestration, we expect agricultural GHG mitigation programs will not be cost-efficient in these areas. Use of either of these practices may be beneficial for other economic or environmental reasons in these regions, but operating a program with the sole objective of mitigating or sequestering GHG emissions is likely to be an unproductive and costly endeavor.

Note, many of the counties with high expected sequestration values and negative predicted rates of adoption for cover-cropping, in bright red in Fig. 5, also have high rates of sequestration and intermediate or high rates of adoption in tillage reduction practices, in maroon and dark purple in Fig. 4. If a program were to incentivize using practices jointly in such counties, it is possible the net return of adopting both practices could become positive despite the high cost of cover-cropping practices. Even though the incentive required might be larger than what is necessary to incentivize tillage reduction in isolation, the cumulative return in carbon sequestration could offset this additional cost given the high sequestration values for both practices in the region.

For both practices, counties with high predicted rates of adoption and high expected carbon sequestration values are concentrated in the Mississippi River Basin, especially in areas near confluences of the Mississippi Rivers and its major tributaries. The higher rate of change suggests producers would require small incentives to adopt the practices, and the greater sequestration increases the marginal benefit from such expenditures. For tillage reductions, the greatest concentrations of counties with these characteristics lie within the Chesapeake Bay watershed or the Upper Mississippi and Lower Missouri sub-basins of the Mississippi River. For cover-cropping, counties with high rates of adoption and net sequestration values are more densely concentrated near the main stem of the Mississippi River by comparison.

An ongoing debate is whether to compensate producers by using payment-for-practice (such as in EQIP) or payment-for-sequestration based on the predicted amount of sequestration (such as in the voluntary carbon market). Our results provide insights on where farmers would be more likely to prefer one type of payment. In general, we anticipate that producers in regions with low net sequestration values will prefer payment-for-practice programs, and producers in areas with greater sequestration will prefer payment-for-sequestration programs. But the incentives will need to be larger to encourage adoption in a payment-for-sequestration program due to the transaction costs involved in estimating sequestration, so the preference for payment-for-sequestration will likely be strongest in the areas we identify as having greater sequestration and high rates of adoption.

## Conclusion

Due to the lack of site-specific estimates on carbon sequestration and producers' costs of adopting conservation practices, using benefit-cost ratios to target expenditures on agricultural GHG mitigation programs is often infeasible. In this paper, we present an alternative approach that can improve targeting utilizing aggregate data that is publicly available. Using a conceptual model of technology adoption, we demonstrated how the pace of adoption in a region serves as a proxy for the risk of non-additional expenditures and the cost of adoption. To anticipate the risk of inefficient expenditures facing U.S. agricultural conservation programs, we predicted county-level rates of adoption for two of the primary changes to agricultural land management currently incentivized by public and private programs: cover-cropping and reductions in tillage. After combining the predicted rates of adoption for cover-cropping and tillage reduction practices with the net change in GHG emissions expected due to their use, we illustrated how the interaction between these two factors will determine the cost-efficiency of voluntary programs intending to mitigate GHG emissions through agricultural land use changes across the U.S.

In regions with high expected sequestration and a greater rate of adoption for conservation practices, producers are more likely to accept a small incentive to adopt a practice and sequester a greater quantity of carbon. However, the greater rate of adoption also suggests an increased risk of incentives going to non-additional adopters, producers who would have used the practice without the incentive. As such, we cannot conclusively state that voluntary agricultural GHG mitigation programs located in counties with high predicted rates of change in practice use and large sequestration will always be comparatively more efficient based on our analysis. Instead, our analysis highlights the challenges to cost-efficiency involved with operating a voluntary agricultural GHG mitigation program in three other conditions.

When the rate of adoption is negative and GHG mitigation benefits are small in magnitude, as is the case for tillage reductions in the Atlantic Plains, producers will likely require large incentives to use the practice which result in minimal mitigation benefits. The expenditures needed to encourage adoption may be smaller in areas where the rate of adoption is high and the change in net GHG emissions is similarly small, but the small marginal benefit to net GHG emissions and greater risk of non-additional adoption will detract from any savings on incentives. Last, for areas with negative rates of adoption and high net GHG emissions reductions, the primary obstacle to cost-efficiency will be the large incentives necessary to make using practices profitable for producers.

One limitation of this work is our reliance on two datapoints from 2012 and 2017 for predicting the rate of change in the acres using cover cropping and tillage reduction practices between 2017 and 2022. As additional time series data on the use of cover-cropping and tillage practices become available, we expect future work will be able to predict adoption trends with greater accuracy and at a finer resolution. Further research would help to understand how more detailed information, such as the types of producers who adopt conservation practices or the likelihood of adoption persisting across years, could be used to refine our approach and test the underlying assumptions. It would be especially valuable to compare the cost of using the approach described in this work against alternative approaches to inferring producers’ willingness to accept with greater transaction costs, such as a reverse auction. Finally, predicting changes in sequestration at fine spatial scales remains an ongoing field of research. Incorporating updated estimates as they emerge and addressing the magnitude of their accompanying uncertainties will improve the utility of similar efforts going forward.

### Supplementary Information

**Additional file 1.** Description of file—**Figure S1.** Change in share of cropland acreage employing cover-cropping between 2012 and 2017. Counties with missing data in either 2012 or 2017 are depicted in dark grey. **Figure S2.** Change in share of cropland acreage using no-till between 2012 and 2017. Counties with missing data in either 2012 or 2017 are depicted in dark grey. **Figure S3.** Change in share of cropland acreage using conservation tillage between 2012 and 2017. **Figure S4.** Change in share of cropland acreage using either no-till or conservation tillage between 2012 and 2017. **Figure S5.** Average carbon sequestration for COMET Planner scenarios involving a reduction in tillage intensity (CPS numbers 329 and 345). **Figure S.6.** Average carbon sequestration for COMET Planner scenarios involving cover-cropping (CPS number 340). **Figure S7.** Variance estimates for random forest predictions of the change in acreage using tillage reduction practices between 2017 and 2022. **Figure S8.** Variance estimates for random forest predictions of the change in acreage cover cropping between 2017 and 2022. **Figure S9.** Predicted rate of change in acres using tillage reduction practices between 2017 and 2022. **Figure S10.** Predicted rate of change in acres using cover cropping practices between 2017 and 2022. **Figure S11.** Variable importance plot for the random forest predicting the county-level rate of change in acreage using a reduced tillage practice. **Figure S12.** Variable importance plot for the random forest predicting the county-level rate of change in acreage using cover crops. **Figure S13.** Average carbon sequestration due to tillage reduction practices by tercile. Values are the average of CPS 329 and 345 practices from COMET Planner. **Figure S14.** Predicted rate of adoption between 2017 and 2022 for tillage reduction practices by category. Rates are divided into those below zero, between 0 and median positive predicted rate, and values above the median positive predicted rate.

## Data Availability

The data supporting the findings of this article were drawn from the following resources available in the public domain: Data on use of cover-cropping, tillage reduction practices, and total cropland acreage are freely available via the Quick Stats database from the United States Department of Agriculture National Agricultural Statistics Service at https://data.nal.usda.gov/dataset/nass-quick-stats; the total area for each county was calculated using the TIGER/Line shapefiles published by the U.S. Census Bureau at https://www.census.gov/geographies/mapping-files/time-series/geo/tiger-line-file.html; the NRCS program obligations for voluntary agricultural conservation programs were downloaded using the NRCS Financial Assistance Program Data Dashboards at https://www.farmers.gov/data/financial-assistance-download; the full COMET-Planner dataset containing county-level estimates of the changes in net GHG emissions for various practices is available for download at http://comet-planner.com/; spatially explicit soil characteristics were created by querying the Soil Data Access website at https://sdmdataaccess.nrcs.usda.gov; the gridded temperature and precipitation data are freely available from the PRISM Climate Group at https://prism.oregonstate.edu/; and the Cultivated Layer from the USDA National Agricultural Statistics Service Cropland Data Layer used to filter the PRISM and SSURGO data was downloaded from https://nassgeodata.gmu.edu/CropScape/.

## Abbreviations

GHG: Greenhouse gas
PES: Payment for Ecosystem Services
EQIP: Environmental Quality Incentives Program
USDA: United States Department of Agriculture
NASS: National Agricultural Statistics Service
NRCS: Natural Resource Conservation Service
CPS: Conservation Practice Standard
SSURGO: Soil Survey Geographic Database

## Declarations

### Authors' Information

The findings and conclusions in this manuscript are those of the authors and should not be construed to represent any official USDA or U.S. government determination or policy. This research was supported in part by U.S. Department of Agriculture's Economic Research Service.
