# Supplementary material for: Predicting the spatial variation in cost-efficiency for agricultural greenhouse gas mitigation programs in the U.S
Source: Carbon Balance Manag. 2024 Feb 9;19:6. doi: 10.1186/s13021-024-00252-6 (PMC10858497; doi:10.1186/s13021-024-00252-6)
Supplement: Supplementary file 1 — Additional file 1. Description of file—Figure S1. Change in share of cropland acreage employing cover-cropping between 2012 and 2017. Counties with missing data in either 2012 or 2017 are depicted in dark grey. Figure S2. Change in share of cropland acreage using no-till between 2012 and 2017. Counties with missing data in either 2012 or 2017 are depicted in dark grey. Figure S3. Change in share of cropland acreage using conservation tillage between 2012 and 2017. Figure S4. Change in share of cropland acreage using either no-till or conservation tillage between 2012 and 2017. Figure S5. Average carbon sequestration for COMET Planner scenarios involving a reduction in tillage intensity (CPS numbers 329 and 345). Figure S.6. Average carbon sequestration for COMET Planner scenarios involving cover-cropping (CPS number 340). Figure S7. Variance estimates for random forest predictions of the change in acreage using tillage reduction practices between 2017 and 2022. Figure S8. Variance estimates for random forest predictions of the change in acreage cover cropping between 2017 and 2022. Figure S9. Predicted rate of change in acres using tillage reduction practices between 2017 and 2022. Figure S10. Predicted rate of change in acres using cover cropping practices between 2017 and 2022. Figure S11. Variable importance plot for the random forest predicting the county-level rate of change in acreage using a reduced tillage practice. Figure S12. Variable importance plot for the random forest predicting the county-level rate of change in acreage using cover crops. Figure S13. Average carbon sequestration due to tillage reduction practices by tercile. Values are the average of CPS 329 and 345 practices from COMET Planner. Figure S14. Predicted rate of adoption between 2017 and 2022 for tillage reduction practices by category. Rates are divided into those below zero, between 0 and median positive predicted rate, and values above the median positive predicted rate. [file 13021_2024_252_MOESM1_ESM.docx]

**Additional file 1**


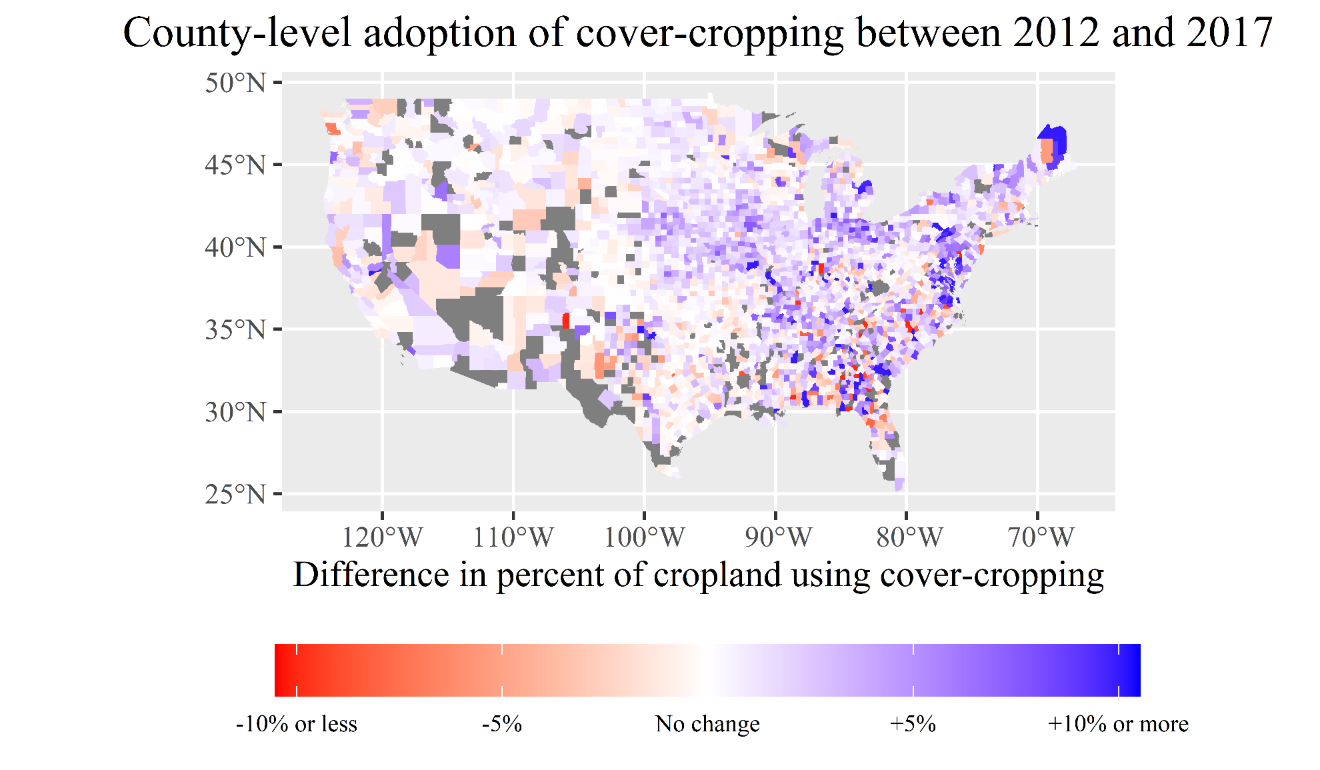


Figure S.1: Change in share of cropland acreage employing cover-cropping between 2012 and 2017. Counties with missing data in either 2012 or 2017 are depicted in dark grey.


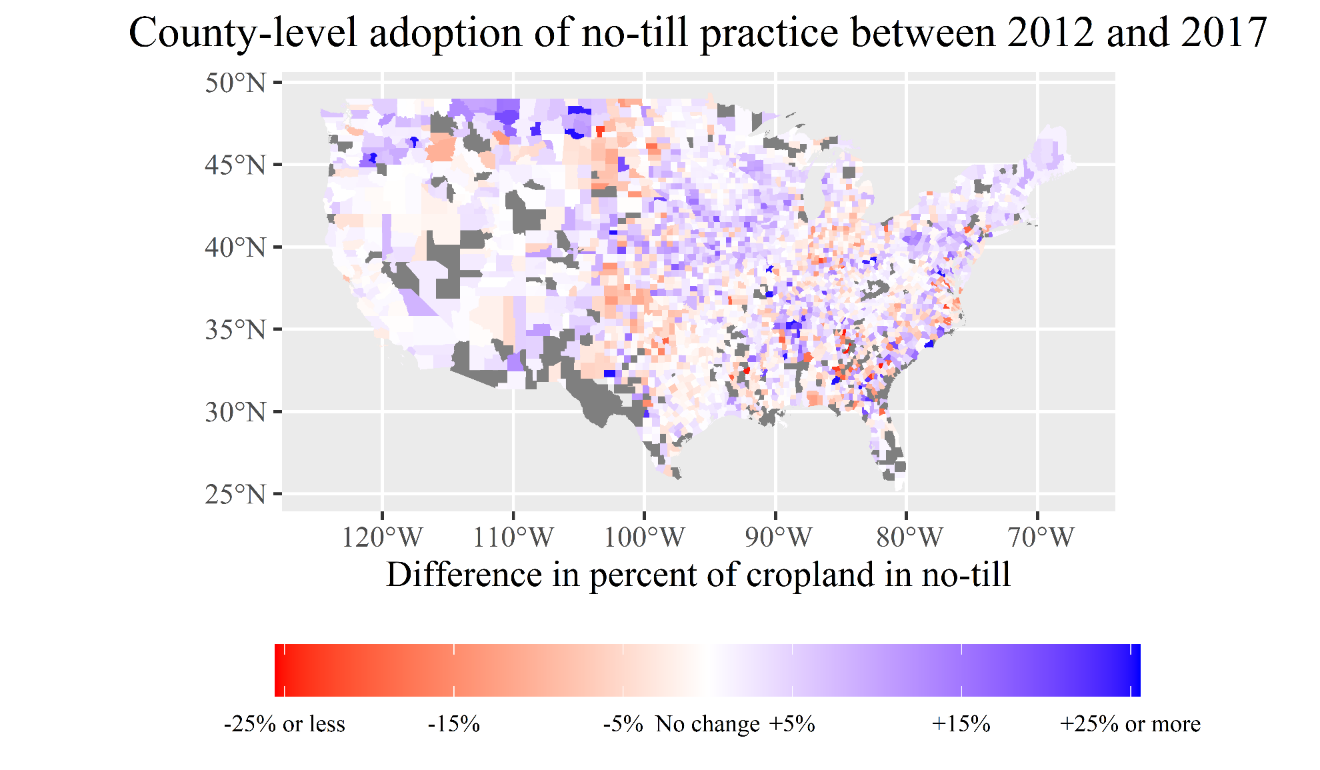


Figure S.2: Change in share of cropland acreage using no-till between 2012 and 2017. Counties with missing data in either 2012 or 2017 are depicted in dark grey.


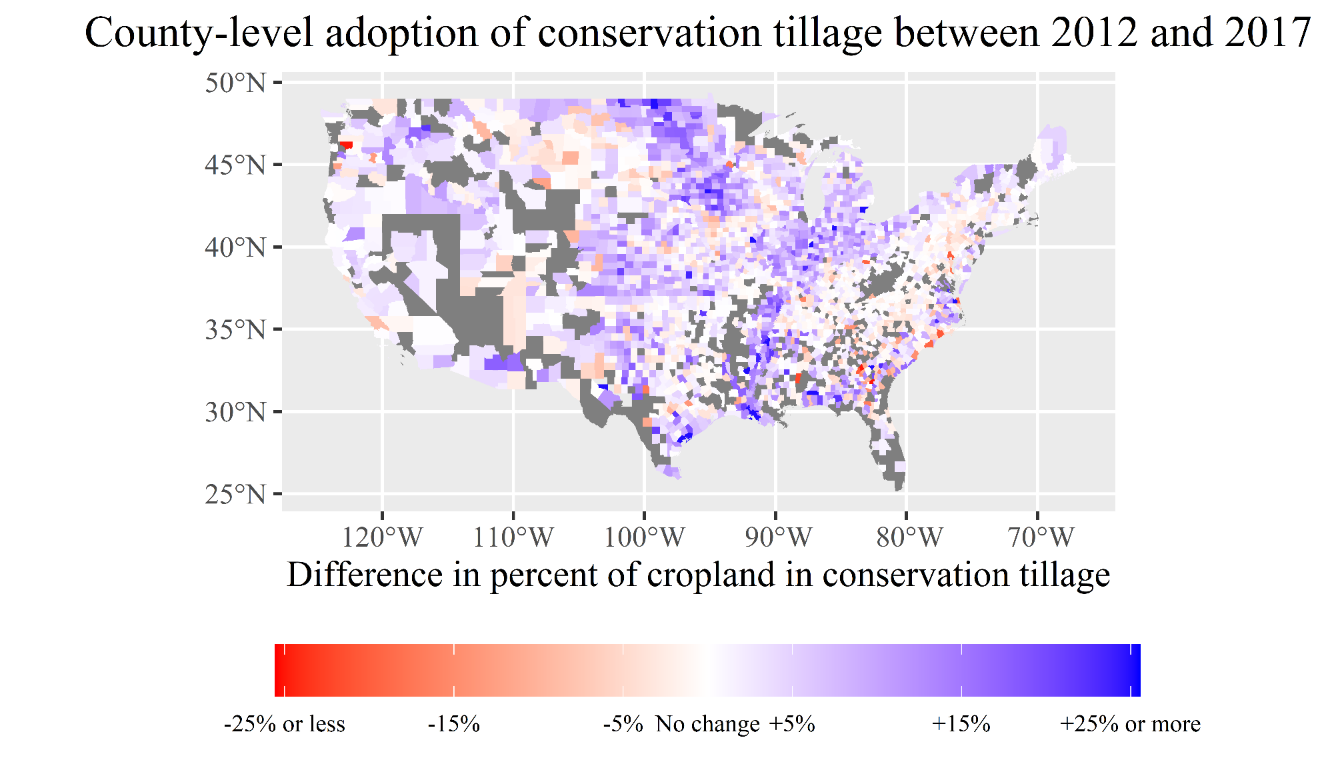


Figure S.3: Change in share of cropland acreage using conservation tillage between 2012 and 2017.


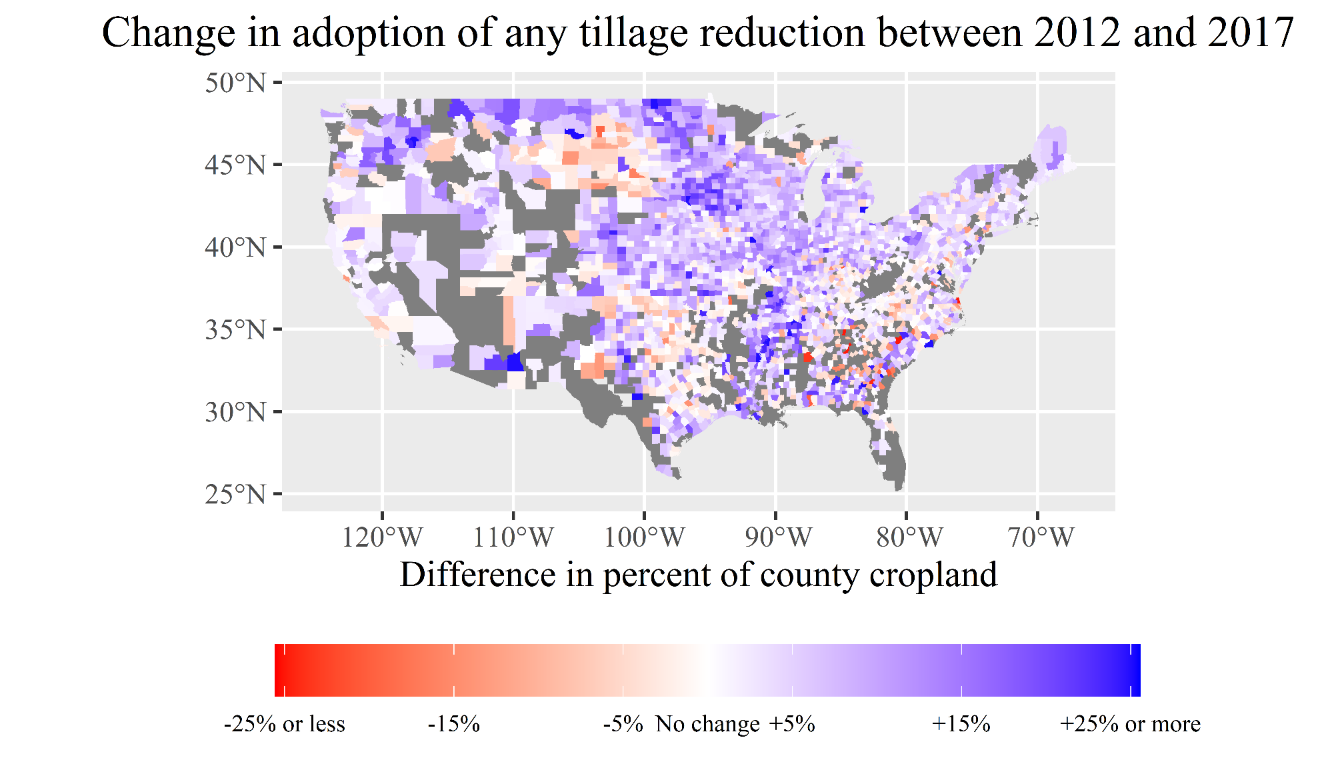


Figure S.4: Change in share of cropland acreage using either no-till or conservation tillage between 2012 and 2017.


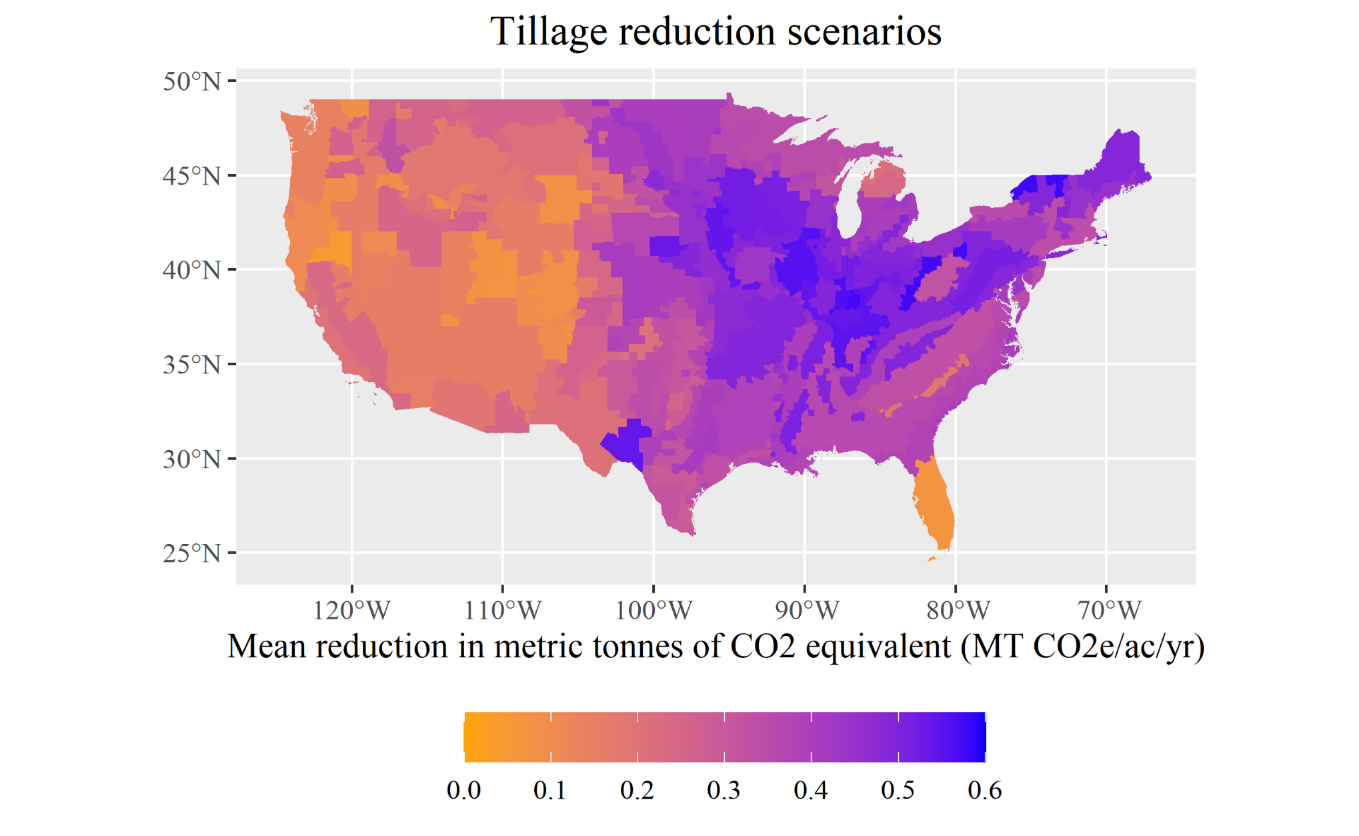


Figure S.5: Average carbon sequestration for COMET Planner scenarios involving a reduction in tillage intensity (CPS numbers 329 and 345).


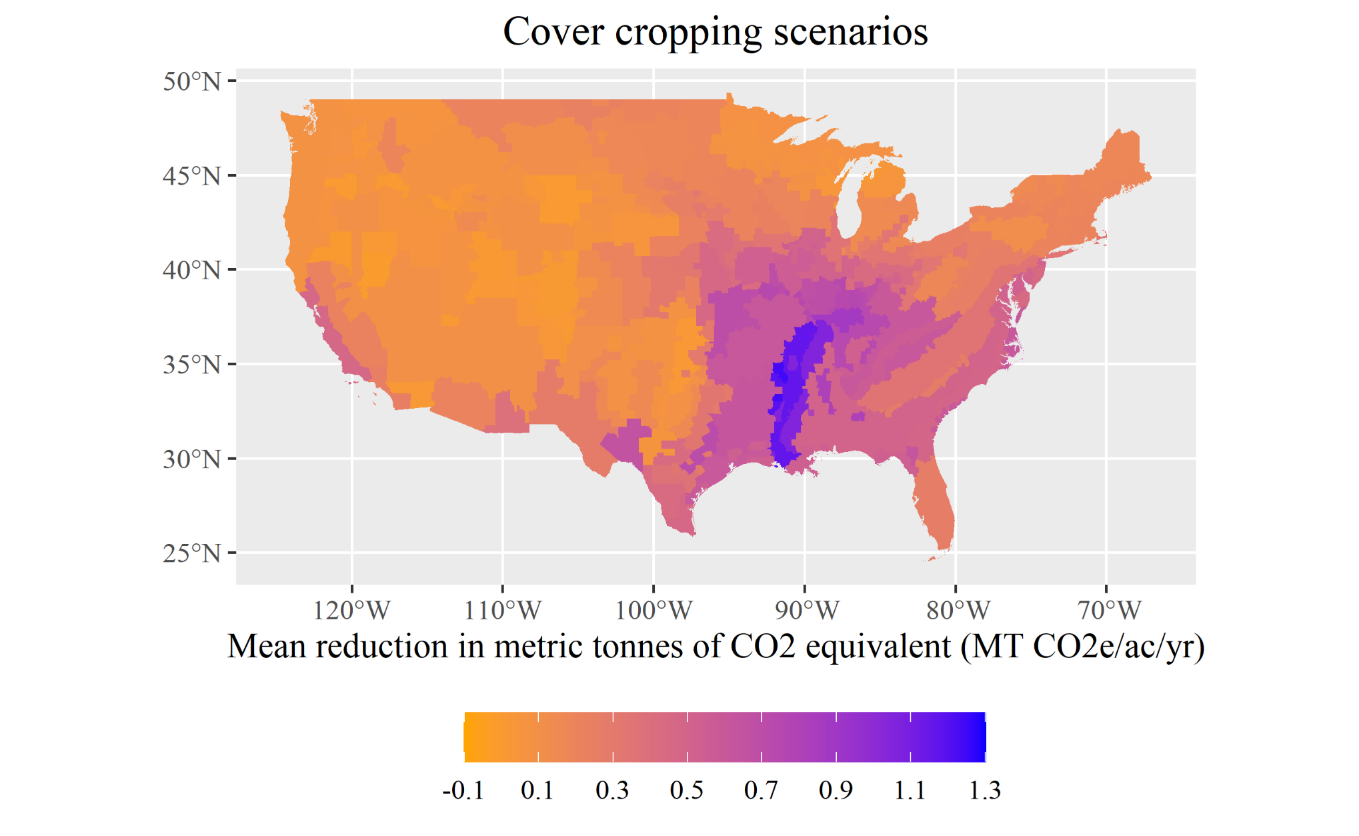


Figure S.6: Average carbon sequestration for COMET Planner scenarios involving cover-cropping (CPS number 340).


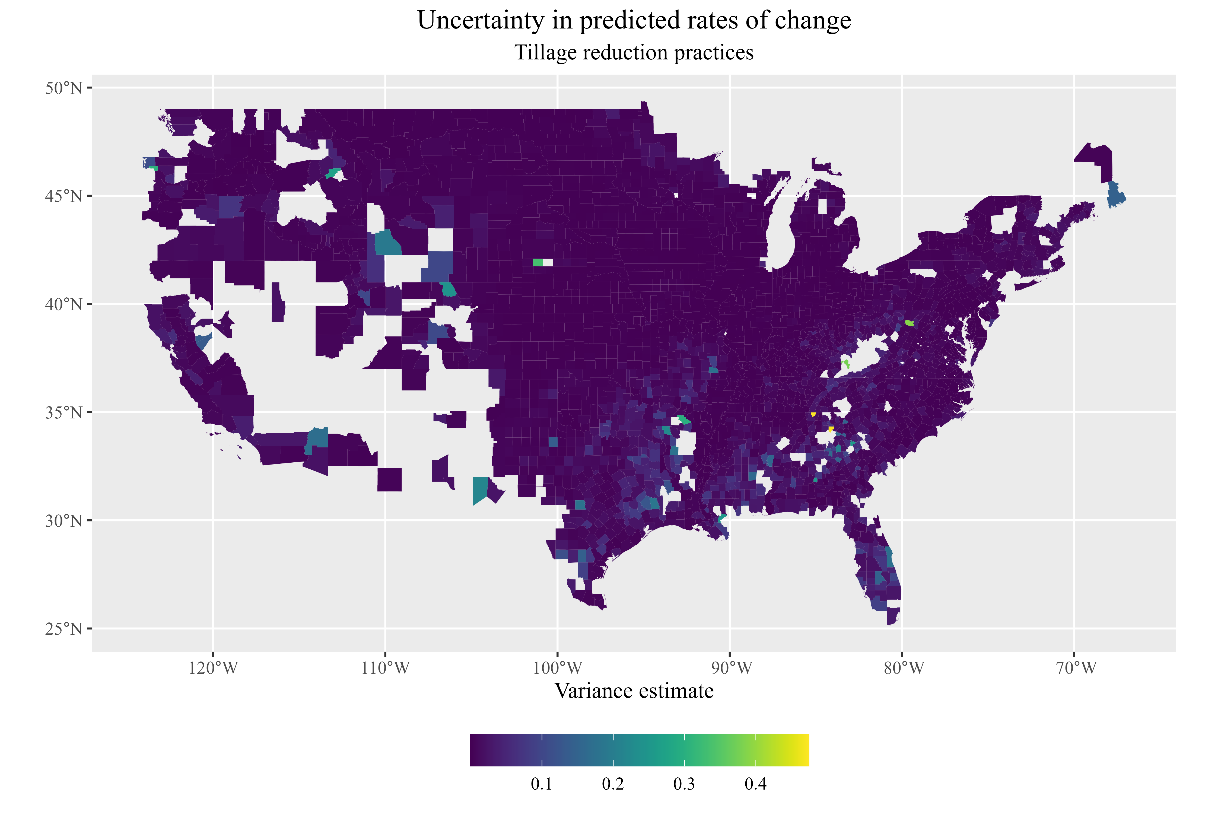


Figure S.7: Variance estimates for random forest predictions of the change in acreage using tillage reduction practices between 2017 and 2022.


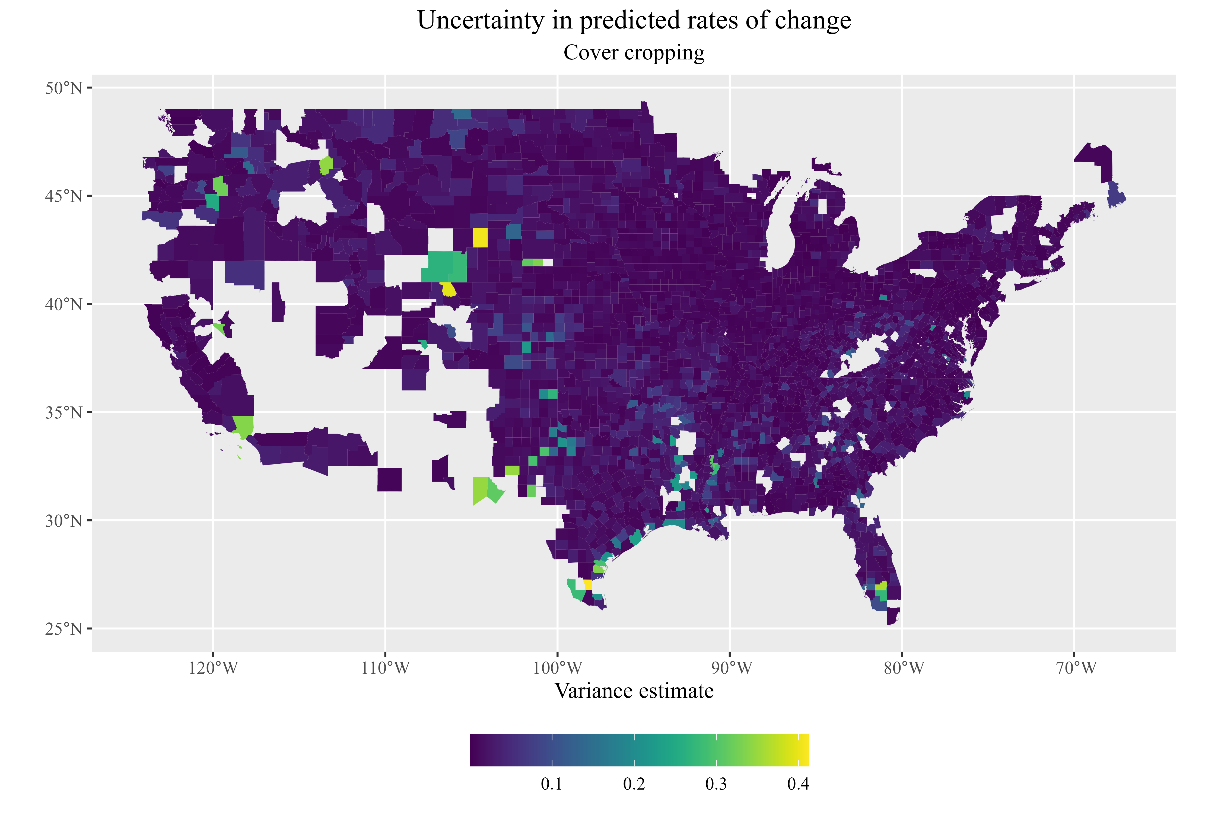


Figure S.8: Variance estimates for random forest predictions of the change in acreage cover cropping between 2017 and 2022.


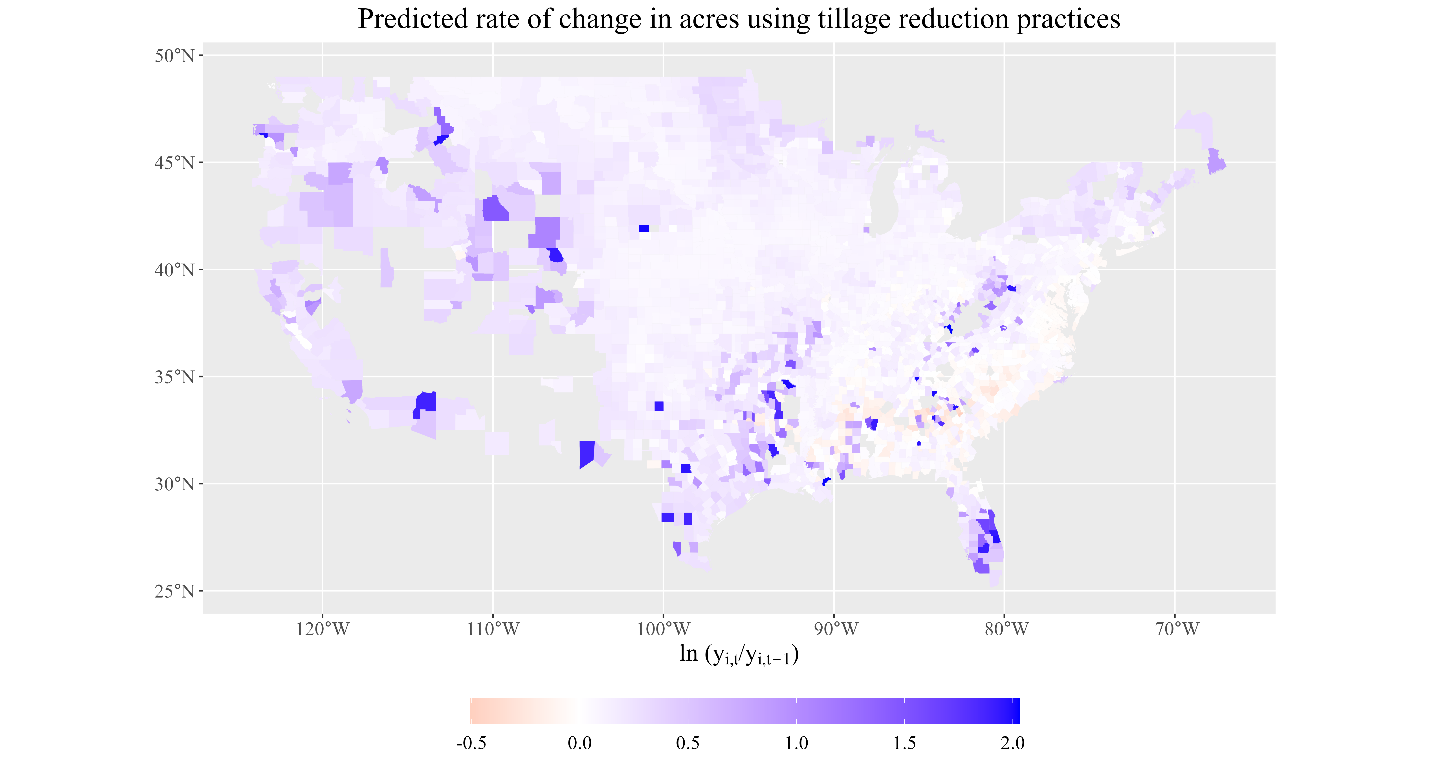


Figure S.9: Predicted rate of change in acres using tillage reduction practices between 2017 and 2022.


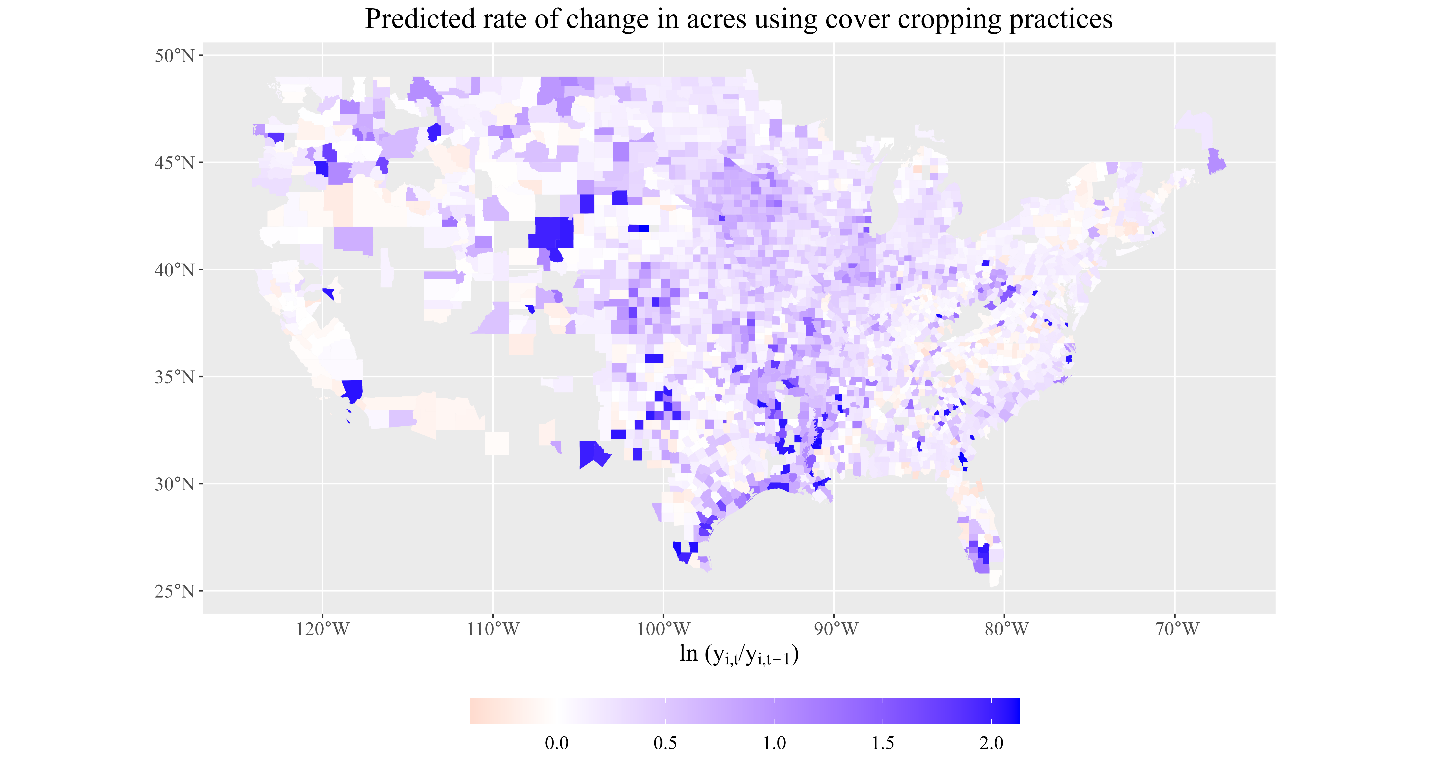


Figure S.10: Predicted rate of change in acres using cover cropping practices between 2017 and 2022.


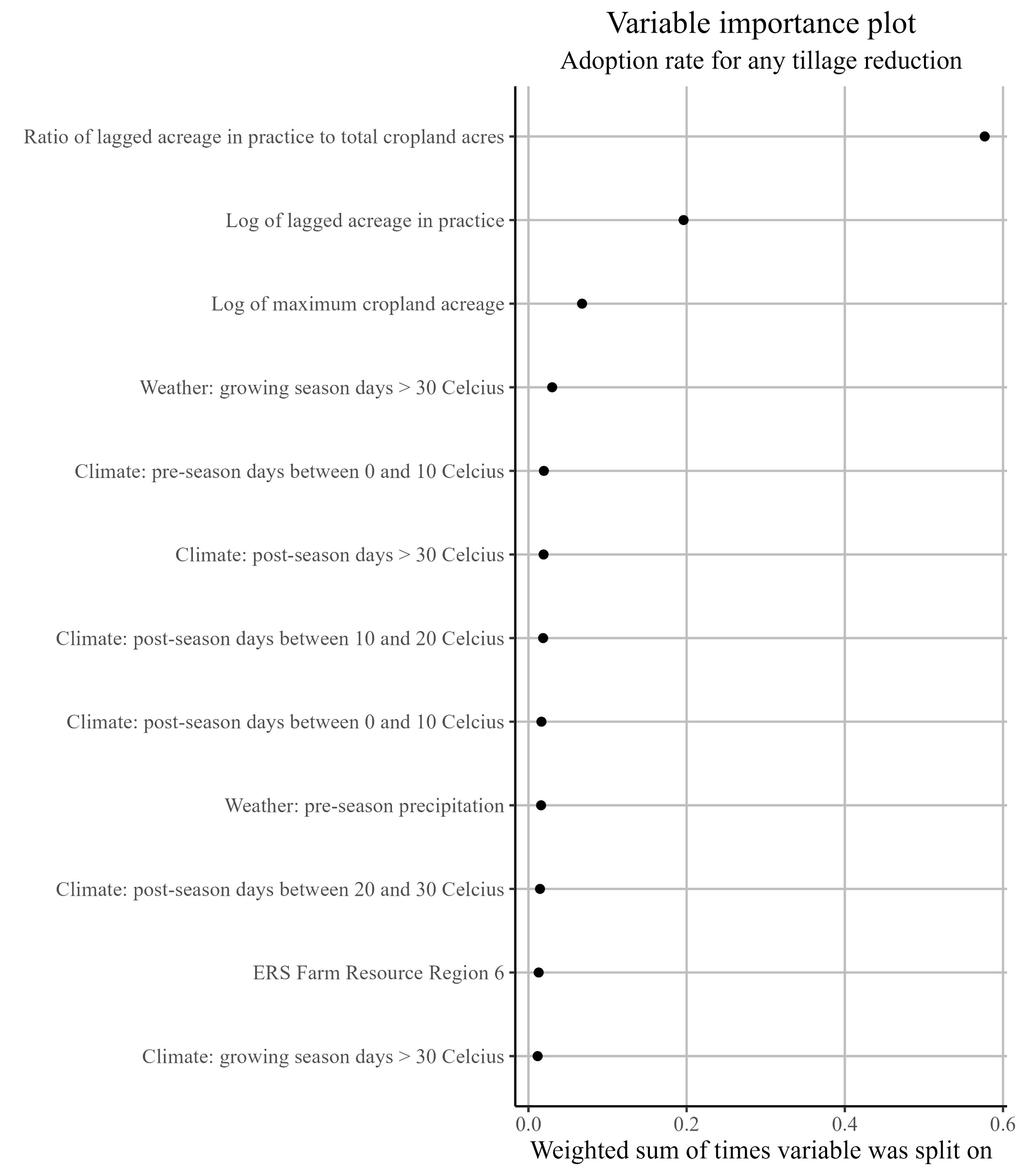


Figure S.11: Variable importance plot for the random forest predicting the county-level rate of change in acreage using a reduced tillage practice.


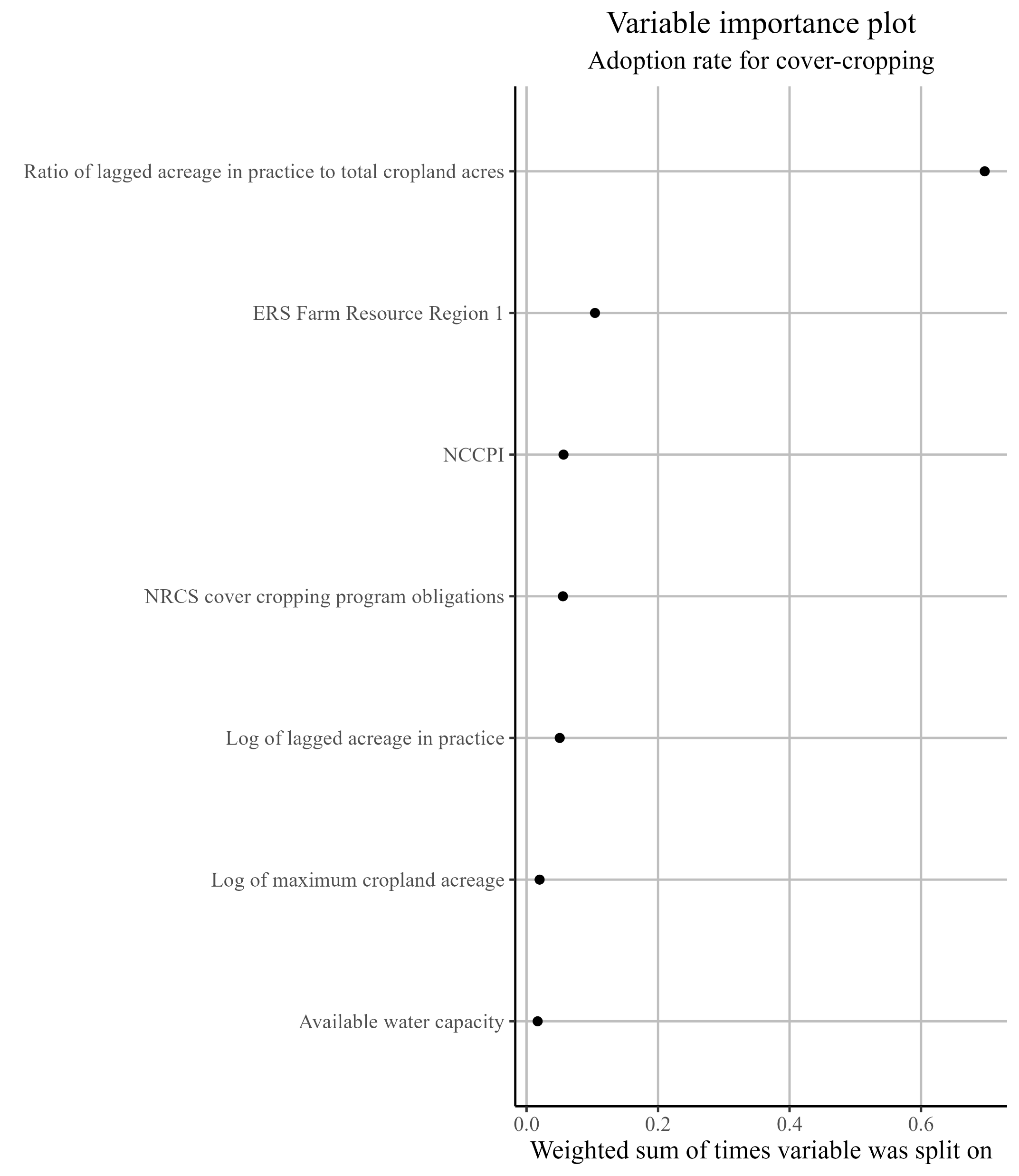


Figure S.12: Variable importance plot for the random forest predicting the county-level rate of change in acreage using cover crops.


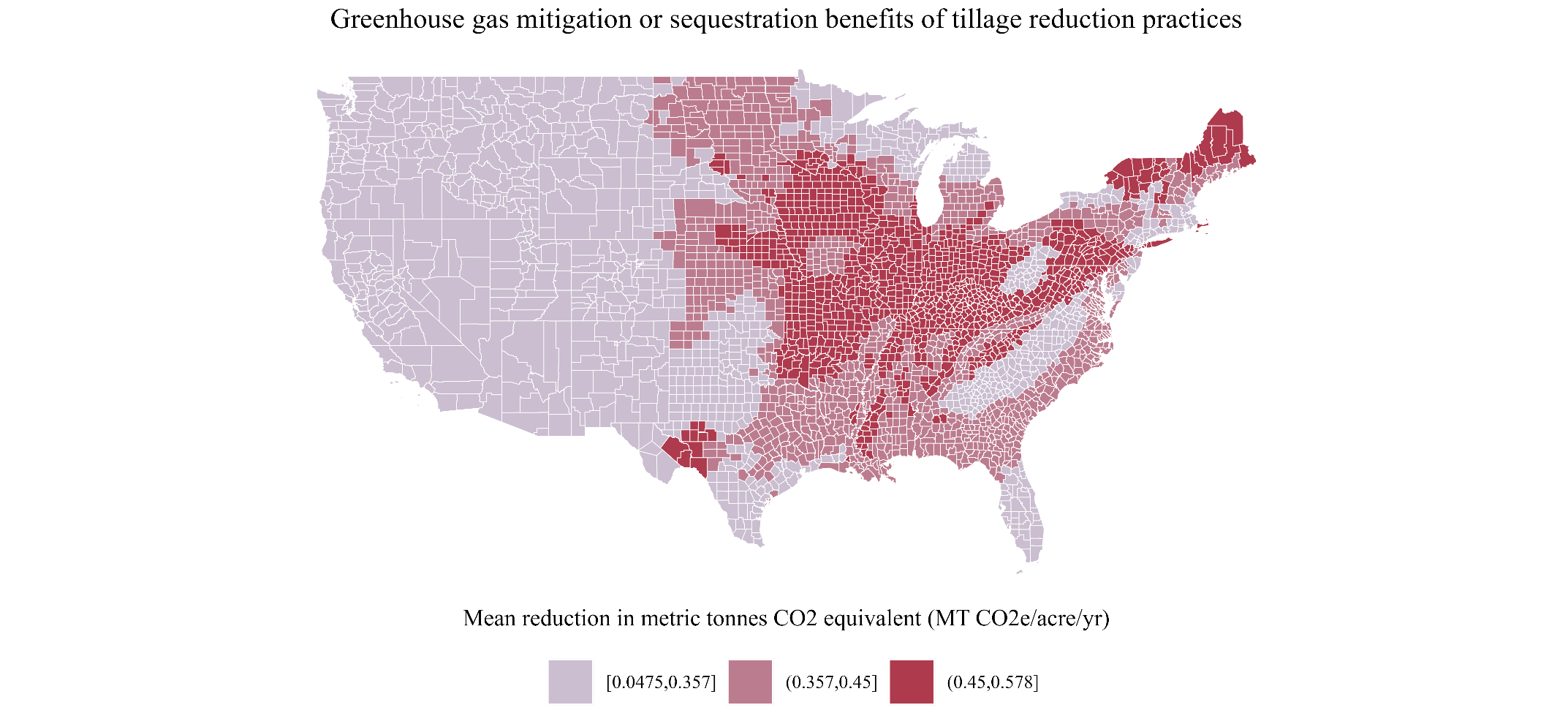


Figure S.13: Average carbon sequestration due to tillage reduction practices by tercile. Values are the average of CPS 329 and 345 practices from COMET Planner.


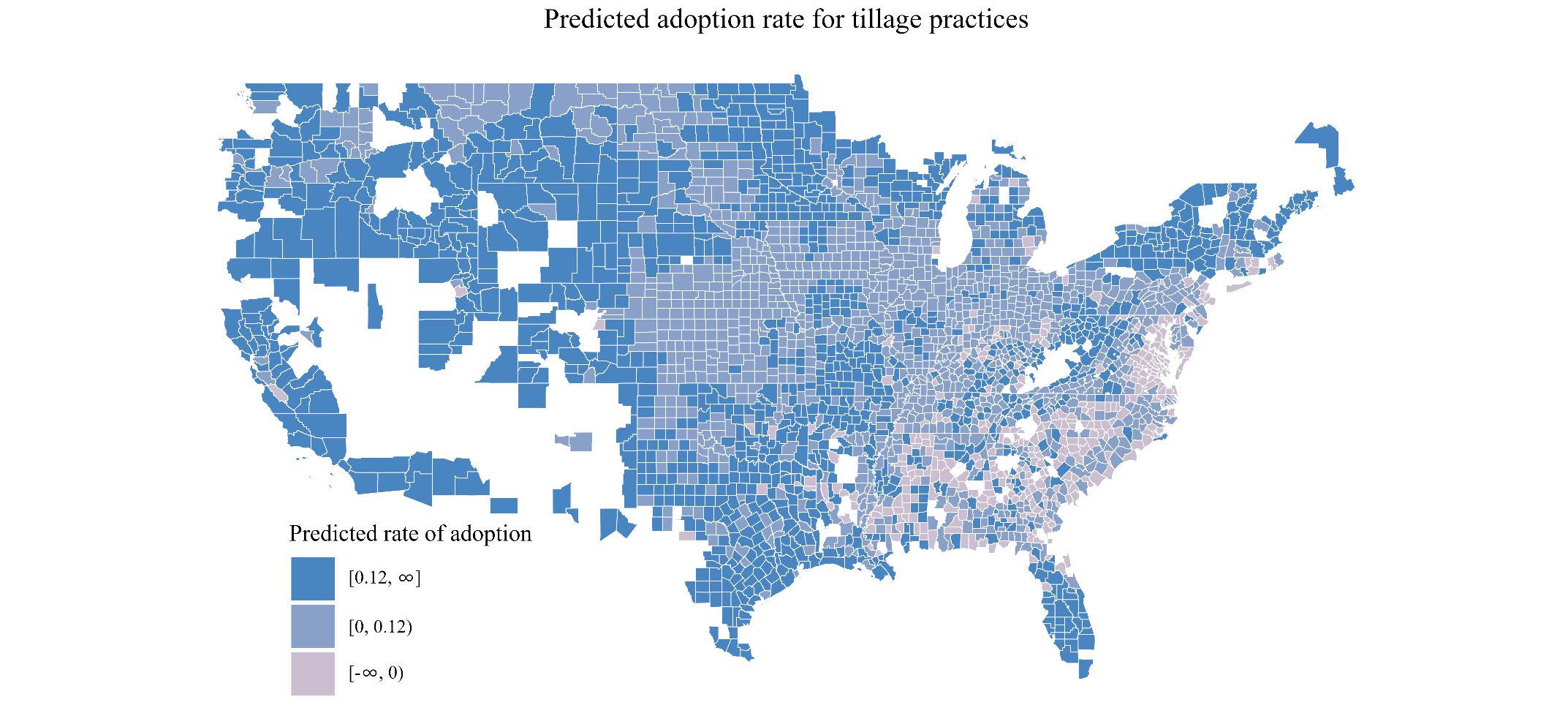


Figure S.14: Predicted rate of adoption between 2017 and 2022 for tillage reduction practices by category. Rates are divided into those below zero, between 0 and median positive predicted rate, and values above the median positive predicted rate.
